# Synthesis and Evaluation of Novel Nitric Oxide-Donating Ligustrazine Derivatives as Potent Antiplatelet Aggregation Agents

**DOI:** 10.3390/molecules28083355

**Published:** 2023-04-11

**Authors:** Han-Xu Li, Jian-Hui Tian, Hua-Yu Li, Xin Wan, Yu Zou

**Affiliations:** Institute of Pharmaceutical Process, Hubei Province Key Laboratory of Occupationl Hazard Identification and Control, School of Medicine, Wuhan University of Science and Technology, Wuhan 430065, China; lihanxu997@sina.com (H.-X.L.);

**Keywords:** ligustrazine, nitric oxide donors, antiplatelet aggregation agents, structure-activity relationships

## Abstract

Antiplatelet aggregation agents have demonstrated clinical benefits in the treatment of ischemic stroke. In our study, a series of novel nitric oxide (NO)-donating ligustrazine derivatives were designed and synthesized as antiplatelet aggregation agents. They were evaluated for the inhibitory effect on 5′-diphosphate (ADP)-induced and arachidonic acid (AA)-induced platelet aggregation in vitro. The results showed that compound **15d** displayed the best activity in both ADP-induced and AA-induced assays, and compound **14a** also showed quite better activity than ligustrazine. The preliminary structure-activity relationships of these novel NO-donating ligustrazine derivatives were discussed. Moreover, these compounds were docked with the thromboxane A2 receptor to study the structure-activity relationships. These results suggested that the novel NO-donating ligustrazine derivatives **14a** and **15d** deserve further study as potent antiplatelet aggregation agents.

## 1. Introduction

Stroke is a highly devastating brain disease and the second leading cause of death across the world. The annual number of cases and deaths due to stroke increased substantially worldwide, thereby resulting in an increasing medical health burden [1]. Among the strokes, ischemic stroke (IS) is a major type commonly caused by thrombus and cerebral arterial stenosis [2]. Despite its difficult treatment, therapeutic approaches against platelet aggregation are still considered a key component of the management of IS, particularly in the secondary prevention of cerebrovascular disease [3,4].

Traditional Chinese medicine is an important source of active natural products, many of which have good therapeutic effects on vascular embolism and tissue ischemia. *Chuanxiong* (*Ligusticum chuanxiong* Hort), a traditional Chinese herbal medicine with a long history of use in China, was first documented in the ancient medical book *ShenNong’s Herbal Classic* as having the effects of promoting blood circulation, dispelling blood stasis and alleviating pain [5]. Moreover, ligustrazine (2,3,5,6-tetramethylpyrazine, TMP), originally a natural product extracted from *chuanxiong*, is made as an injection that is widely used as a clinical drug for occlusive cerebrovascular disease in China [6,7]. TMP has a short half-life in vivo because it is easily metabolized and rapidly excreted, which results in its limited actuation duration [8]. Therefore, a large number of ligustrazine derivatives were synthesized and modified to improve the effects and pharmacokinetic properties in the past few decades.

On the other hand, nitric oxide (NO), a well-known gaseous messenger molecule, plays an important role in regulating physiological functions in cerebrovascular systems [9]. In cardiovascular and cerebrovascular systems, NO, produced by both endothelial cells and platelets, has important antithrombotic effects by decreasing platelet activation [10]. It is difficult to control NO gas when directly used in vivo, while NO donors’ organic nitrate esters can release NO into the body via enzymatic or nonenzymatic pathways. Therefore, organic nitrate esters can be used to produce the pharmacological effects of NO in vivo, such as inhibiting platelet aggregation and thrombus formation. Furthermore, connecting various NO donors to cardiovascular and cerebrovascular disease drugs was a great strategy for enhancing their therapeutic activities and/or mitigating their adverse effects [11,12,13].

As mentioned, ligustrazine derivatives could avoid being rapidly metabolized in vivo and prolong their actuation duration, while the introduction of NO donors to the structure could enhance antiplatelet aggregation activity. Thus, we connected the NO donors with different ligustrazine derivatives via various linkers to generate several novel NO-donating ligustrazine derivatives (Table 1). It was expected that these compounds could exhibit better antiplatelet aggregation activities. Herein, we report the synthesis and biological evaluation of the novel NO-donating ligustrazine derivatives as potent antiplatelet aggregation agents.

## 2. Results and Discussion

### 2.1. Chemistry

In order to obtain the target compounds, two types of ligustrazine intermediates, 2-hydroxymethyl-3,5,6-trimethylpyrazine (**3**) and (E)-3-(3,5,6-trimethylpyrazin-2-yl) acrylic acid (**6**) were selected. Compound **3** is an active metabolite of ligustrazine with antiplatelet aggregation [14]. Compound **6** is an acrylic acid derivative that was initially synthesized for improved solubility and developed more bioactive analogs [15]. Therefore, compounds **3** and **6** were often used as active compound fragments for the synthesis of target active compounds in the previous studies [16,17], as was the case in our study.

**Scheme 1:**
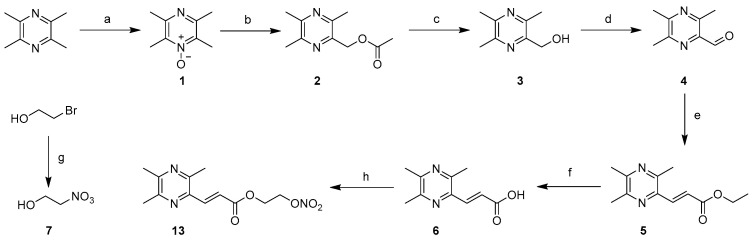
Syntheses of compounds **3**, **6** and **13**. Reagents and conditions: (a) 30%H_2_O_2_, AcOH, 90 °C, 4 h, 69%; (b) Ac_2_O, 130 °C, 2.5 h, 38–52%; (c) 20%NaOH, rt, 12 h, 85–92%; (d) MnO_2_, EtOH, reflux, 6 h, 95%; (e) toluene, NaH, triethyl phosphonoacetate, rt, under dark, 8–10 h, 80%; (f) ① KOH/H_2_O, 16 h, ② HCl/H_2_O, 97%; (g) AgNO_3_, CH_3_CN, 70 °C, under dark, 3 h, 78%; (h) 7, DCC, DMAP, CH_2_Cl_2_, rt, 47%.

We first synthesized the simplest NO-donating ligustrazine derivatives, **13**, by coupling **6** with a NO donor, which was used as the reference compound for the activity study of other compounds. The syntheses of **3**, **6** and **13** are shown in Scheme 1. Commercially available ligustrazine hydrochloride was neutralized with sodium hydroxide to obtain ligustrazine trihydrate. Ligustrazine trihydrate was treated with an acetic anhydride 30% hydrogen dioxide solution in the presence of acetic acid at 90 °C to obtain ligustrazine N-Oxide **1**. Compound **1** underwent a Boekelheide reaction with acetic anhydride at 130 °C to yield **2**, which was hydrolyzed to **3**. The total yield was 33.7%. Compound **6** was smoothly synthesized from **3** via oxidation, the Wittig–Horner reaction and the hydrolysis reaction [18], with a total yield of 71.6%. Compound **7** was obtained via a reaction of 2-bromoethanol with AgNO_3_ in acetonitrile, followed by condensation with **6** to provide **13**.

**Scheme 2:**
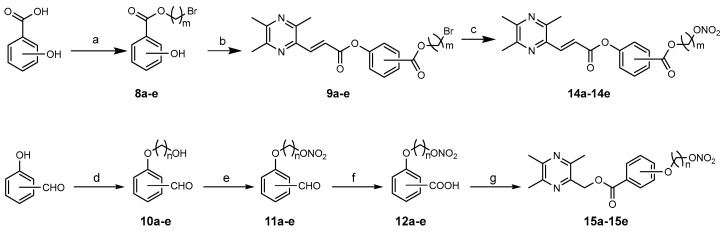
Syntheses of compounds **14a**–**14e** and **15a**–**15e**. Reagents and conditions: (a) Br(CH_2_)_m_Br, Et_3_N, acetone, reflux, 5 h, 29–60%; (b) 6, DCC, DMAP, CH_2_Cl_2_, rt, 70–93%; (c) AgNO_3_, CH_3_CN, 70 °C, under dark, 3 h, 37–64%; (d) Br(CH_2_)_n_OH, K_2_CO_3_, acetone, reflux, 6 h, 29–67%; (e) Fuming HNO_3_, AcOH, Ac_2_O, –5 °C to rt, 2 h, 60–66%; (f) KMnO_4_, acetone, reflux, 2 h, 82–90%; (g) 3, DCC, DMAP, CH_2_Cl_2_, rt, 43–54%.

Derivatives **14a**–**14e** were synthesized from a series of hydroxybenzoic acid derivatives (Scheme 2). O-hydroxybenzoic acid was treated with 1,2-dibromoethane to obtain **8a**, followed by condensation with **6** to provide **9a**. The nitrate ester of **14a** was obtained from a reaction of **10a** with AgNO_3_ in acetonitrile. The preparation of **14b**–**14e** followed the same route. Further, **15a**–**15e** were synthesized from a series of hydroxybenzaldehyde derivatives [19]. First, hydroxybenzaldehyde reacted with the corresponding brominated alcohol to give **10a**–**e**. Then, compounds **11a**–**e** were converted to **12a**–**e** by nitration and oxidation. Finally, condensation of **12a**–**e** with **3** in the presence of N,N′-dicyclohexylcarbodimide (DCC) and 4-dimethylaminopyridine (DMAP) formed **15a**–**15e**. Except for compounds **15a**–**15c**, separated by thin-layer chromatography, the other compounds were purified by column chromatography. All the structures were identified by ^1^H NMR, ^13^C NMR and HRMS.

### 2.2. In Vitro Antiplatelet Aggregation

**Figure 1:**
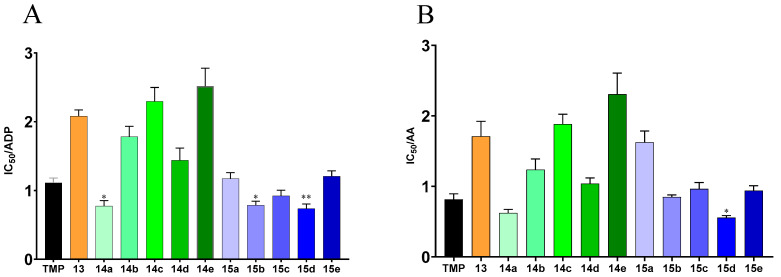
The effect of target compounds on platelet aggregation in vitro. (**A**) The IC_50_ values of target compounds against 10 μmol·L^−1^ ADP-induced rabbit platelet aggregation in vitro; (**B**) The IC_50_ values of target compounds against 1 mmol·L^−1^ AA-induced rabbit platelet aggregation in vitro (mean ± SD, n = 5, * *p* < 0.05, ** *p* < 0.01 vs. TMP).

We evaluated the inhibitory effects of all the target compounds on platelet aggregation induced by adenosine 5′-diphosphate (ADP) (10 μmol·L^−1^) and arachidonic acid (AA) (1 mmol·L^−1^) in rabbit platelet plasma using the Born turbidimetric method. The results, shown in Figure 1, showed that compound **15d** displayed the best activity in the ADP-induced and AA-induced assays (IC_50_ = 0.7347 mM and 0.5565 mM, respectively), both of which were more active than TMP under the same conditions.

Firstly, we connected **6** with Aspirin and the NO donor to generate compound **14a**. We expected that the compound could be hydrolyzed in vivo by esterases to release ligustrazine, Aspirin and NO, which would enhance the inhibitory effects on platelet aggregation due to synergism. To probe the effect of the substitution position on activity, we designed **14b** and **14c**. In addition, an ether bond was applied to connect the NO donor with aromatic acid to enhance the stability and liposolubility of a fragment of the NO donor. Thus, we designed the second series of compounds **15a**–**15c**. After these compounds were tested for activities, the best active compound of each type was selected, and we increased the length of the linker next to the NO donor in the structure to search for an appropriate length of the carbon chain. Finally, we synthesized and tested target compounds **14d**, **14e**, **15d** and **15e**.

Among compounds **14a**–**14e**, **14a** has the best activity in ADP-induced and AA-induced assays, with IC_50_ values of 0.7736 mM and 0.6236 mM. Compared with **13**, which only contained ligustrazine moiety and NO donor moiety in the structure, most of the compounds that add in an Aspirin fragment show a stronger inhibitory effect in two assays, which indicates that the inhibitory activity is not strongly increased due to the direct addition of the NO donor, and the presence of the Aspirin fragment enhances the activity against platelet aggregation. The activities of compounds **14a**–**14e** were significantly affected by the substitution position and length of the carbon chain of the NO-donor linker. On the one hand, as with the substitution position of the hydroxyl group in Aspirin, the compound with the NO-donor linker in the ortho position has the best activity in all assays. This further demonstrates the effect of Aspirin fragments on antiplatelet aggregation activity. On the other hand, their inhibitory activities are markedly affected by the length of the carbon chain of the NO donor. The inhibitory activity of compounds **14d** and **14e** decreases significantly when the linker length is increased.

It is **15d** that represents the best activity among compounds **15a**–**15e**. The compounds **15a**–**15c** were first synthesized and assayed to evaluate the optimal substitution position of the NO-donor linker. The result showed that meta-substituted **15b** displayed the optimal inhibitory activity in both assays before we increased the length of the carbon chain of the NO donor in the structure. However, the length of the carbon chain had an unremarkable effect on the improvement in inhibitory activity according to the change in the IC_50_ values of compounds **15d** and **15e**. Nevertheless, most of the compounds of **15a**–**15e** displayed better activity than compounds **14a**–**14e**. As mentioned above, the preliminary structure-activity relationship revealed that the compound derivatives that differed in the composition of the active structure fragment, substituent position and length of the carbon chain of the NO-donor linker showed varied activities against platelet aggregation.

### 2.3. Molecular Docking

The thromboxane A2 receptor (TBXA2R) plays a key role in physiological processes, such as platelet aggregation and the contraction of vascular smooth muscle cells; thus it is often used as an important target in the study of drugs for the treatment of cardiovascular and cerebrovascular diseases, such as atherosclerosis and cerebral ischemia [20]. Based on the characteristics of this receptor, this study docked with the designed compound at the active site.

**Figure 2:**
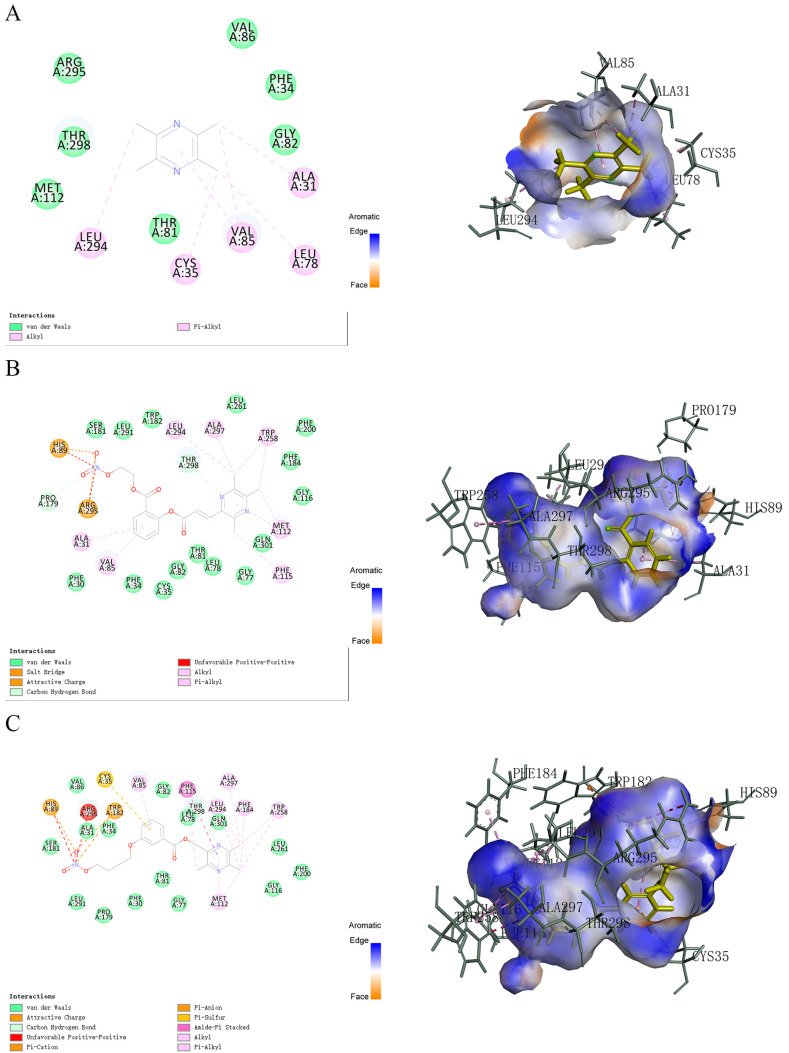
(**A**) TMP, (**B**) **14a** and (**C**) **15d** and interactions with TBXA2R (PDB 6IIV).

The results of docking efficiency (-CDOCKER_INTERACTION_ENERGY) were basically consistent with the results of the corresponding cell experiments, especially for compounds **15a**–**15e** (Table S2). It was found that the main forms of the interactions between TMP and proteins are Pi-alkyl and hydrophobic bonds of alkyl, and the interactions between TMP and proteins only occur on the surface layer of the active site instead of in the depths of the protein void, resulting in low docking efficiency (Figure 2A). In contrast, the newly designed ligustrazine derivative increased the length of the molecule, allowing the compound structure to penetrate deeper into the protein cavity at the active site and interact with it. For example, the results of the interaction between **14a** and TBXA2R showed that the changes in the structure and volume of the compound promoted deeper binding between **14a** and the protein cavity, and the types and number of non-bond interactions between **14a** and TBXA2R were more abundant (Figure 2B). By comparing the results of the interactions between **14a**, **14b** and **14c** with the receptor, instead of para-substituted **14c** at the active site showing an obvious linear structure extending inwardly, ortho-substituted compound **14a**, at the active site, showed a larger number and types of beneficial interactions with the receptor, which increased the interaction intensity. In the structure of compounds **15a**–**15e**, the benzene ring and pyrazine rings were relatively close together, but they had a greater number of non-bond interactions with the acceptor, which seemed to suggest that the existence of a double bond between aryl groups in the structure of compounds **14a**–**14e** was not appropriate (Figure 2C). Furthermore, a 2D interaction diagram analysis further showed that there was less interaction between the active site of the receptor and the carbon chain of the NO donor, which pointed out that the length of the carbon chain could not have a positive effect on the interaction between the compound and the receptor.

Based on the above results, we speculated that the mode of action of the compounds might be to inhibit AA-induced platelet aggregation by acting on the active site of TBXA2R. The Aspirin structure contributed more to the binding between the compounds and TBXA2R, while the length of the linking carbon chain produced a greater effect on the stability of the NO donor and release of NO. In addition, it is likely that various compositions of fragment molecules and NO-donor linkers may have different effects on the liposolubility and metabolism of target compounds, leading to different inhibitory activities.

## 3. Materials and Methods

### 3.1. Chemistry

All chemicals were of analytical reagent grade, and all solvents were of reagent grade. The reactions were monitored by thin-layer chromatography (TLC) (Qingdao Haiyang Chemical Co., Qingdao, China). The solutions containing the products were concentrated with a rotary evaporator. Flash column chromatography was performed in a column packed with 200–300 mesh silica gel. TLC was performed on silica gel GF254-coated glass sheets, and the spots were visualized under UV light (254 nm). NMR spectra were recorded with the Agilent DD2 600 MHz NMR spectrometer (Agilent Technologies Inc., Santa Clara, CA, USA) in CDCl_3_ containing 0.03% *v/v* tetramethylsilane (TMS) as the internal standard. The HRMS was recorded on a Xevo G2-XS QTOF instrument (Waters, MA, USA).

#### 3.1.1. General Procedure for the Preparation of **1**

NaOH (8.0 g, 200 mmol) and ligustrazine hydrochloride (41.8 g, 200 mmol) were dissolved in 300 mL and 100 mL of water at 0 °C to prepare solutions, respectively. To the stirred solution of ligustrazine hydrochloride was added slowly the NaOH solution. After stirring for one hour, water was removed under reduced pressure, and the product was collected by filtration and dried by a standard method to obtain ligustrazine trihydrate. Then, a solution of ligustrazine trihydrate (20.4 g, 107 mmol) in glacial acetic acid (30 mL) was added to the 30% H_2_O_2_ aqueous solution (12.1 mL, 107 mmol). The reaction mixture was stirred at 90 °C for 2 h before another portion of the 30% H_2_O_2_ aqueous solution (12.1 mL, 107 mmol) was added and stirred at 90 °C for another 2 h. The resultant mixture was cooled to room temperature, alkalized to pH = 10 with 50% sodium hydroxide and extracted with dichloromethane (150 mL, 50 mL × 3). The organic layer was combined and dried with anhydrous sodium sulfate for 8 h and evaporated in vacuo to afford ligustrazine mono-N-oxide (**1**).

#### 3.1.2. General Procedure for the Preparation of **3**

Compound **1** (13.8 g, 90.6 mmol) was treated with acetic anhydride (17.1 mL, 181.2 mmol) at 130 °C for 2.5 h. The mixture was added in 20% NaOH (aq, 100 mL) and stirred at room temperature overnight. The resultant mixture was extracted with dichloromethane (160 mL, 40 mL × 4). The extract was dried with anhydrous sodium sulfate for 8 h and evaporated in vacuo to obtain the crude product. The crude product was recrystallized from a mixed solvent of petroleum ether (PE) and ethyl acetate (EA) (PE/EA = 10/1, *v*/*v*) to obtain **3** as a yellow solid (5.5 g), an overall yield of 33.7%.

#### 3.1.3. General Procedure for the Preparation of **4**

To a solution of **3** (6.09 g, 40 mmol) in anhydrous ethanol (20 mL), active MnO_2_ was added (20.8 g, 240 mmol). After heating to reflux for 6 h, the reaction mixture was filtered through a sintered glass funnel layered with a layer of diatomite, and the filtrate was evaporated in vacuo to obtain 3,5,6-trimethylpyrazine-2-carbaldehyde (**4**) as a yellow solid (5.67 g).

#### 3.1.4. General Procedure for the Preparation of **5**

To a solution of compound **4** (5.5 g, 36.6 mmol) in toluene (50 mL), NaH (1.76 g, 73.2 mmol) was added carefully in an ice bath, and after 0.5 h, triethyl phosphonoacetate (9.0 g, 40.3 mmol) was added dropwise, and the mixture was stirred at room temperature overnight. The reaction solution was diluted by adding EtOAc (50 mL), then washed with saturated brine (50 mL × 3), and the combined extract was dried over anhydrous sodium sulfate for 8 h and concentrated in vacuo. The crude product was purified by column chromatography (PE/EA = 8/1, *v/v*) to obtain **5** as a pale-yellow solid (6.45 g).

#### 3.1.5. General Procedure for the Preparation of **6**

Compound **5** (6.45 g, 29.3 mmol) was dissolved in a mixed solvent of tetrahydrofuran (THF) and water (THF/H_2_O = 2/1, *v/v*, 30 mL), and then sodium hydroxide (2.35 g, 58.6 mmol) was slowly added, and the mixture was stirred at room temperature for 4 h. The reaction mixture was adjusted with 2 mol/L of hydrochloric acid to pH = 4, followed by the addition of sodium chloride solid until it dissolved more. Next, the mixture was extracted with EtOAc (90 mL, 30 mL × 3), and the combined organic phase was dried over anhydrous sodium sulfate for 8 h and evaporated in vacuo to obtain **6** as a white solid (5.5 g).

#### 3.1.6. General Procedure for the Preparation of **7**

2-Bromoethanol (1.25 g, 10 mmol) was dissolved in acetonitrile (50 mL), and AgNO_3_ (2.55 g, 15 mmol) was added to the reaction solution, which was then stirred under darkness at 70 °C for 3 h. After cooling, the reaction mixture was filtered to remove the solid product, and the filtrate was concentrated in vacuo. The concentrate was stirred for 10 min after adding 30 mL EtOAc, then filtered and concentrated again to obtain **7** (1.0 g) as a yellow liquid.

#### 3.1.7. General Procedure for the Preparation of **8a**–**e**

The corresponding dibromoethane (7.5 mmol,1.5 eq) and triethylamine (7.5 mmol, 1.5 eq) were added to a solution of hydroxybenzoic acid (0.69 g, 5 mmol) in acetone (20 mL). After heating to reflux for 6 h under nitrogen, the reaction mixture was cooled to room temperature and evaporated in vacuo. The residue was stirred for 5 min after adding 30 mL of EtOAc and then filtered and concentrated. The concentrate was purified by column chromatography (PE/EA = 10/1, 8/1, *v/v*) to obtain **8a**–**e**: **8a**, colorless oil, PE/EA = 8/1, yield 22%; **8b**, colorless oil, PE/EA = 8/1, yield 27%; **8c**, white solid, PE/EA = 8/1, yield 29%; **8d**, colorless oil, PE/EA = 10/1, yield 61%; **8e**, colorless oil, PE/EA = 10/1, yield 60%.

#### 3.1.8. General Procedure for the Preparation of **9a**–**e**

DCC (0.23 g, 1.1 mmol) and **6** (0.19 g, 1.0 mmol) were dissolved in anhydrous dichloromethane (20 mL), and a catalytic amount of DMAP was added to the reaction solution, which was then stirred for about 15 min. Next, **8a**–**e** (1.1 mmol,1.1 eq) was added to the solution and stirred for 4 h at room temperature. The resultant mixture was filtered, and the filtrate was concentrated in vacuo. The concentrate was purified by column chromatography (PE/EA = 5/1, 4/1, *v/v*) to obtain **9a**–**e**: **9a**, white powder, PE/EA = 5/1, yield 67%; **9b**, white powder, PE/EA = 5/1, yield 70%; **9c**, white powder, PE/EA = 5/1, yield 70%; **9d**, yellow solid, PE/EA = 5/1, yield 72%; **9e**, white solid, PE/EA = 5/1, yield 93%.

#### 3.1.9. General Procedure for the Preparation of **10a**–**e**

To a solution of hydroxybenzaldehyde (1.22 g, 10 mmol) in acetone (20 mL), the corresponding brominated alcohol (20 mmol, 2.0 eq) and anhydrous K_2_CO_3_ (20 mmol, 2.0 eq) were added. After heating to reflux for 6 h under nitrogen, the reaction mixture was cooled to room temperature and concentrated in vacuo. The concentrate was purified by column chromatography (PE/EA = 4/1, 2/1, *v/v*) to obtain **10a**–**e**: **10a**, yellow oil, PE/EA = 4/1–2/1, yield 38%; **10b**, cream oil, PE/EA = 4/1, 2/1, yield 29%; **10c**, mauve oil, PE/EA = 3/1, yield 67%; **10d**, yellow oil, PE/EA = 4/1, yield 35%; **10e**, yellow oil, PE/EA = 4/1, yield 58%.

#### 3.1.10. General Procedure for the Preparation of **11a**–**e**

Compounds **10a**–**e** (1.0 eq) and a small amount of carbamide were dissolved in glacial acetic acid (5 mL), and mixtures of Fuming HNO_3_ (5.0 eq) and acetic anhydride (5 mL) were slowly added dropwise at −5 °C. The reaction solution was stirred at −5 °C for 0.5 h and then kept at room temperature for 2 h. The resultant solution was poured into ice water, then adjusted to pH = 7 with the saturated NaHCO_3_ solution and extracted with EtOAc (60 mL, 20 mL × 3). The combined organic layer was washed with brine, dried over anhydrous sodium sulfate for 8 h and concentrated in vacuo. The concentrate was purified by column chromatography (PE/EA = 4/1, 3/1, *v/v*) to obtain **11a**–**e**: **11a**, yellow oil, PE/EA = 4/1, yield 66%; **11b**, yellow oil, PE/EA = 4/1, yield 63%; **11c**, yellow oil, PE/EA = 3/1, yield 60%; **11d**, yellow oil, PE/EA = 4/1, yield 61%; **11e**, yellow oil, PE/EA = 4/1, yield 63%.

#### 3.1.11. General Procedure for the Preparation of **12a**–**e**

KMnO_4_ (1.5 eq) was added to a solution of **11a**–**e** (1.0 eq) in acetone (20 mL) and heated to reflux for 2 h. The reaction mixture was added to the saturated NaHSO_3_ solution until its red faded completely. Then, the pH of the solution was adjusted to 5 with 2 mol/L of hydrochloric acid, and after being filtered through a sintered glass funnel layered with a layer of diatomite, the filtrate was extracted with EtOAc (60 mL, 20 mL × 3). The combined organic extracts were washed with brine, dried over anhydrous sodium sulfate for 8 h and evaporated in vacuo to afford **12a**–**e**: **12a**, yellow oil, yield 82%; **12b**, yellow solid, yield 88%; **12c**, white solid, yield 90%; **12d**, yellow oil, yield 86%; **12e**, yellow oil, yield 85%.

#### 3.1.12. 2-(Nitrooxy)ethyl (E)-3-(3,5,6-Trimethylpyrazin-2-yl)acrylate (**13**)

Both **6** (0.74 g, 3.8 mmol) and DCC (0.79 g, 3.8 mmol) were dissolved in anhydrous dichloromethane (30 mL), and a catalytic amount of DMAP was added to the reaction solution, which was then stirred for about 15 min. Next, compound **7** (0.38 g, 3.5 mmol) was added to the solution and stirred for 6 h at room temperature. The resultant mixture was filtered, and the filtrate was concentrated in vacuo. The concentrate was stirred for 15 min after adding 20 mL EtOAc, then filtered and concentrated again, and was purified by column chromatography (PE/EA = 5/1, *v/v*) to obtain target compound **13** (0.40 g), with an overall yield of 41%. White solid, ^1^H NMR (600 MHz, Chloroform-*d*): δ 7.88 (d, 1H, *J* = 15.3 Hz, ArCH = CH), 7.06 (d, 1H, *J* = 15.3 Hz, ArCH = CH), 4.79–4.72 (m, 2H, CH_2_CH_2_NO_3_), 4.55–4.48 (m, 2H, CH_2_CH_2_NO_3_), 2.62 (s, 3H, ArCH_3_), 2.52 (d, 6H, *J* = 4.6 Hz, ArCH_3_); ^13^C NMR (151 MHz, CDCl_3_) δ 166.28, 153.01, 150.02, 149.12, 142.25, 140.06, 121.87, 77.20, 76.99, 76.77, 70.44, 60.36, 22.02, 21.69, 20.66; HRMS (ESI): m/z: 281.1112 calc. for C_12_H_15_N_3_O_5_ [M + H]^+^, found 281.1109, ppm error −1.1.

#### 3.1.13. General Procedure for the Preparation of **14a**–**14e**

**9a**–**e** (1.0 eq) was dissolved in acetonitrile (20 mL), and AgNO_3_ (1.5 eq) was added to the reaction solution, which was then stirred under darkness at 70 °C for 3 h. After cooling, the reaction mixture was filtered to remove the solid product, and the filtrate was concentrated in vacuo. The concentrate was stirred for 10 min after adding 30 mL of EtOAc, then filtered and concentrated again, and purified by column chromatography (PE/EA = 4/1, 2/1, *v/v*) or TLC (DCM/MeOH = 50/1, *v/v*) to obtain target compounds **14a**–**14e**.

2-(nitrooxy)ethyl (E)-2-((3-(3,5,6-trimethylpyrazin-2-yl)acryloyl)oxy)benzoate (**14a**). Cream solid, PE/EA = 2/1, yield 37%; ^1^H NMR (600 MHz, Chloroform-*d*): δ 8.08 (d, 1H, *J* = 6.4 Hz, 6-ArH), 8.05 (d, 1H, *J* = 15.6 Hz, ArCH = CH), 7.60 (t, 1H, *J* = 7.8 Hz, 4-ArH), 7.36 (t,1H, *J* = 7.6 Hz, 5-ArH), 7.31 (d, 1H, *J* = 15.3 Hz, ArCH = CH), 7.19 (d, 1H, *J* = 8.0 Hz, 3-ArH), 4.38 (t, 2H, *J* = 4.5 Hz, CH_2_CH_2_NO_3_), 3.84 (t, 2H, *J* = 4.5 Hz, CH_2_CH_2_NO_3_), 2.66 (s, 3H, ArCH_3_), 2.56 (d, 6H, *J* = 5.9 Hz, ArCH_3_); ^13^C NMR (151 MHz, CDCl_3_) δ 165.28, 164.98, 152.75, 150.56, 150.22, 148.86, 142.79, 140.50, 133.99, 132.03, 126.19, 123.78, 123.43, 122.71, 77.22, 77.01, 76.80, 66.93, 60.71, 21.63, 21.61, 20.29; HRMS (ESI): m/z: 402.1301 calc. for C_19_H_20_N_3_O_7_ [M + H]^+^, found 402.1291, ppm error −2.5.

2-(nitrooxy)ethyl (E)-3-((3-(3,5,6-trimethylpyrazin-2-yl)acryloyl)oxy)benzoate (**14b**). Yellow solid, PE/EA = 2/1, yield 64%; ^1^H NMR (600 MHz, Chloroform-*d*) δ 8.03 (d, 1H, *J* = 15.2 Hz, ArCH = CH), 7.96 (d, 1H, *J* = 7.8 Hz, 6-ArH), 7.86 (s, 1H, 2-ArH), 7.49 (m, 1H, *J* = 7.9 Hz, 5-ArH), 7.39 (d, 1H, *J* = 8.1 Hz, 4- ArH), 7.24 (d, 1H, *J* = 15.3 Hz, ArCH = CH), 4.47 (t, 2H, *J* = 4.6 Hz, CH_2_CH_2_NO_3_), 3.96 (t, 2H, *J* = 4.6 Hz, CH_2_CH_2_NO_3_), 2.64 (s, 3H, ArCH_3_), 2.55 (s, 6H, ArCH_3_); ^13^C NMR (151 MHz, CDCl_3_) δ 165.93, 164.95, 153.15, 150.68, 150.26, 149.17, 142.28, 140.95, 131.41, 129.49, 127.12, 126.51, 122.89, 121.88, 77.22, 77.01, 76.80, 66.90, 61.19, 21.95, 21.71, 20.58; HRMS (ESI): m/z: 402.1301 calc. for C_19_H_20_N_3_O_7_ [M + H]^+^, found 402.1300, ppm error −0.2.

2-(nitrooxy)ethyl (E)-4-((3-(3,5,6-trimethylpyrazin-2-yl)acryloyl)oxy)benzoate (**14c**). Yellow solid, PE/EA = 2/1, yield 50%; ^1^H NMR (600 MHz, Chloroform-*d*): δ 8.12 (d, 2H, *J* = 8.5 Hz, 2,6-ArH), 8.03 (d, 1H, *J* = 15.2 Hz, ArCH = CH), 7.31–7.24 (m, 3H, 3,5-ArH and ArCH = CH), 4.48 (t, 2H, *J* = 4.7 Hz, CH_2_CH_2_NO_3_), 3.97 (t, 2H, *J* = 4.6 Hz, CH_2_CH_2_NO_3_), 2.67 (s, 3H, ArCH_3_), 2.58 (d, 6H, *J* = 7.2 Hz, ArCH_3_); ^13^C NMR (151 MHz, CDCl_3_) δ 166.11, 164.47, 154.54, 152.61, 150.83, 148.59, 142.77, 140.60, 131.29, 127.43, 122.34, 121.63, 77.21, 77.00, 76.79, 66.77, 61.33, 21.71, 21.52, 20.12; HRMS (ESI): m/z: 402.1301 calc. for C_19_H_20_N_3_O_7_ [M + H]^+^, found 402.1299, ppm error −0.5.

4-(nitrooxy)butyl (E)-2-((3-(3,5,6-trimethylpyrazin-2-yl)acryloyl)oxy)benzoate (**14d**). Cream solid, PE/EA = 4/1, yield 39%; ^1^H NMR (600 MHz, Chloroform-*d*) δ 8.06 (d, 1H, *J* = 14.7 Hz, ArCH = CH), 8.04 (d, 1H, *J* = 7.9 Hz, 6-ArH), 7.60 (t, 1H, *J* = 7.8 Hz, 4-ArH), 7.36 (t, 1H, *J* = 7.7 Hz, 5-ArH), 7.32 (d, 1H, *J* = 15.2 Hz, ArCH = CH), 7.19 (d, 1H, *J* = 8.1 Hz, 3-ArH), 4.43 (t, 2H, *J* = 5.9 Hz, CH_2_NO_3_), 4.30 (t, 2H, *J* = 5.6 Hz, COOCH_2_), 2.65 (s, 3H, ArCH_3_), 2.55 (d, 6H, *J* = 5.6 Hz, ArCH_3_), 1.87–1.77 (m, 4H, CH_2_CH_2_CH_2_CH_2_); ^13^C NMR (151 MHz, CDCl_3_) δ 165.17, 164.62, 153.10, 150.39, 150.23, 149.14, 142.28, 140.79, 133.91, 131.78, 126.11, 123.84, 123.52, 122.00, 77.22, 77.01, 76.79, 72.53, 64.18, 24.99, 23.62, 21.97, 21.70, 20.58; HRMS (ESI): m/z: 430.1614 calc. for C_21_H_24_N_3_O_7_ [M + H]^+^, found 430.1617, ppm error 0.7.

6-(nitrooxy)hexyl (E)-2-((3-(3,5,6-trimethylpyrazin-2-yl)acryloyl)oxy)benzoate (**14e**). Yellow oil, DCM/MeOH = 50/1, yield 59%; ^1^H NMR (600 MHz, Chloroform-*d*) δ 8.01 (d, 1H, *J* = 15.3 Hz, ArCH = CH), 8.00 (d, 1H, *J* = 8.5 Hz, 6-ArH), 7.55 (t, 1H, *J* = 7.7 Hz, 4-ArH), 7.31 (t, 1H, *J* = 6.8 Hz, 5-ArH), 7.29 (d, 1H, *J* = 15.4 Hz, ArCH = CH), 7.15 (d, 1H, *J* = 8.1 Hz, 3-ArH), 4.34 (t, 2H, *J* = 6.6 Hz, CH_2_NO_3_), 4.22 (t, 2H, *J* = 6.6 Hz, COOCH_2_), 2.61 (s, 3H, ArCH_3_), 2.51 (d, 6H, *J* = 4.1 Hz, ArCH_3_), 1.65 (td, 4H, *J* = 14.5, 7.6 Hz, CH_2_CH_2_CH_2_CH_2_CH_2_CH_2_), 1.37 (td, 4H, *J* = 11.7, 10.4, 6.8 Hz, CH_2_CH_2_CH_2_CH_2_CH_2_CH_2_); ^13^C NMR (151 MHz, CDCl_3_) δ 165.15, 164.69, 153.08, 150.38, 150.08, 149.15, 142.27, 140.63, 133.71, 133.68, 131.78, 131.74, 126.04, 126.02, 123.81, 123.79, 123.77, 122.12, 122.09, 77.27, 77.06, 76.85, 73.06, 65.07, 64.92, 28.44, 28.39, 26.55, 25.62, 25.54, 25.29, 25.17, 22.03, 22.01, 21.73, 21.69, 20.67, 20.65; HRMS (ESI): m/z: 458.1927 calc. for C_23_H_28_N_3_O_7_ [M + H]^+^, found 458.1927, ppm error 0.0.

#### 3.1.14. General Procedure for the Preparation of **15a**–**15e**

Compound **12a**–**e** (1.2 mmol, 1.2 eq) and DCC (1.2 mmol,1.2 eq) were dissolved in anhydrous dichloromethane (20 mL), and a catalytic amount of DMAP was added to the reaction solution, which was then stirred for about 15 min. Next, **3** (1.0 mmol,1.0 eq) was added to the solution and stirred for 6 h at room temperature. The resultant mixture was filtered, and the filtrate was concentrated in vacuo. The concentrate was purified by TLC (PE/EA/EtOH = 30/10/1, *v/v/v*) to obtain the target compounds **15a**–**15e**.

(3,5,6-trimethylpyrazin-2-yl)methyl 2-(2-(nitrooxy)ethoxy)benzoate (**15a**). Yellow solid, 43% yield; ^1^H NMR (600 MHz, Chloroform-*d*): δ 7.85 (d, 1H, *J* = 7.8 Hz, 6-ArH), 7.47 (t, *J* = 7.8 Hz, 1H, 4-ArH), 7.04 (t, *J* = 7.6 Hz, 1H, 5-ArH), 6.94 (d, 1H, *J* = 8.3 Hz, 3-ArH), 5.42 (s, 2H, ArCH_2_), 4.72 (t, 2H, *J* = 4.7 Hz, CH_2_CH_2_NO_3_), 4.28 (t, 2H, *J* = 4.7 Hz, CH_2_CH_2_NO_3_), 2.59 (s, 3H, ArCH_3_), 2.52 (d, 6H, *J* = 6.9 Hz, ArCH_3_); ^13^C NMR (151 MHz, CDCl_3_) δ 165.54, 157.63, 151.38, 149.24, 148.97, 144.81, 133.69, 132.09, 121.60, 120.81, 114.28, 77.24, 77.02, 76.81, 70.75, 65.70, 65.57, 21.61, 21.36, 20.52; HRMS (ESI): m/z: 362.1352 calc. for C_17_H_20_N_3_O_6_ [M + H]^+^, found 362.1361, ppm error 2.5.

(3,5,6-trimethylpyrazin-2-yl)methyl 3-(2-(nitrooxy)ethoxy)benzoate (**15b**). Cream solid, 54% yield; ^1^H NMR (600 MHz, Chloroform-*d*): δ 7.69 (dd, 1H, *J* = 7.7, 1.4 Hz, 6-ArH), 7.55 (s, 1H, 2-ArH), 7.35 (t, 1H, *J* = 7.9 Hz, 5-ArH), 7.11 (dd, 1H, *J* = 8.3, 2.9 Hz, 4-ArH), 5.44 (s, 2H, ArCH_2_), 4.82 (t, 2H, *J* = 4.6 Hz, CH_2_CH_2_NO_3_), 4.28 (t, 2H, *J* = 4.5 Hz, CH_2_CH_2_NO_3_), 2.59 (s, 3H, ArCH_3_), 2.52 (d, 6H, *J* = 9.8 Hz, ArCH_3_); ^13^C NMR (151 MHz, CDCl_3_) δ 165.81, 157.90, 151.47, 149.26, 149.02, 144.68, 131.19, 129.65, 123.06, 120.22, 114.69, 77.24, 77.03, 76.82, 70.73, 65.93, 64.22, 21.63, 21.37, 20.54; HRMS (ESI): m/z: 362.1352 calc. for C_17_H_20_N_3_O_6_ [M + H]^+^, found 362.1353, ppm error 0.3.

(3,5,6-trimethylpyrazin-2-yl)methyl 4-(2-(nitrooxy)ethoxy)benzoate (**15c**). White solid, 45% yield; ^1^H NMR (600 MHz, Chloroform-*d*): δ 8.01 (d, 2H, *J* = 8.5 Hz, 2,6-ArH), 6.90 (d, 2H, *J* = 8.3 Hz, 3,5-ArH), 5.42 (s, 2H, ArCH_2_), 4.83 (t, 2H, *J* = 4.5 Hz, CH_2_CH_2_NO_3_), 4.30 (t, 2H, *J* = 4.5 Hz, CH_2_CH_2_NO_3_), 2.58 (s, 3H, ArCH_3_), 2.52 (d, 6H, *J* = 8.3 Hz, ArCH_3_); ^13^C NMR (151 MHz, CDCl_3_) δ 165.69, 161.72, 151.33, 149.28, 148.93, 144.93, 131.86, 131.80, 123.06, 114.15, 114.10, 77.26, 77.05, 76.84, 70.57, 65.65, 64.07, 21.63, 21.38, 20.55; HRMS (ESI): m/z: 362.1352 calc. for C_17_H_20_N_3_O_6_ [M + H]^+^, found 362.1351, ppm error −0.3.

(3,5,6-trimethylpyrazin-2-yl)methyl 3-(4-(nitrooxy)butoxy)benzoate (**15d**). Colorless oil, 54% yield; ^1^H NMR (600 MHz, Chloroform-*d*): δ 7.59 (dd, 1H, *J* = 7.7, 1.3 Hz, 6-ArH), 7.49 (s, 1H, 2-ArH), 7.28 (t, *J* = 8.0 Hz, 1H, 5-ArH), 7.04 (dd, 1H, *J* = 8.3, 2.6 Hz, 4-ArH), 5.39 (s, 2H, ArCH_2_), 4.49 (t, 2H, *J* = 6.1 Hz, CH_2_NO_3_), 3.98 (t, 2H, *J* = 5.6 Hz, OCH_2_), 2.54 (s, 3H, ArCH_3_), 2.48 (d, 6H, *J* = 9.2 Hz, ArCH_3_), 1.88 (ddt, 4H, *J* = 10.7, 5.8, 2.2 Hz, CH_2_CH_2_CH_2_CH_2_); ^13^C NMR (151 MHz, CDCl_3_) δ 166.03, 158.69, 151.42, 149.31, 148.99, 144.79, 131.06, 129.48, 122.33, 119.98, 114.77, 77.22, 77.01, 76.79, 72.79, 67.13, 65.92, 25.46, 23.80, 21.65, 21.40, 20.57; HRMS (ESI): m/z: 390.1665 calc. for C_19_H_24_N_3_O_6_ [M + H]^+^, found 390.1668, ppm error 0.8.

(3,5,6-trimethylpyrazin-2-yl)methyl 3-((6-(nitrooxy)hexyl)oxy)benzoate (**15e**). Colorless oil, 49% yield; ^1^H NMR (600 MHz, Chloroform-*d*) δ 7.58 (dd, 1H, *J* = 7.7, 1.4 Hz, 6-ArH), 7.50 (s, 1H, 2-ArH), 7.27 (t, 1H, *J* = 8.0 Hz, 5-ArH), 7.04 (dd, 1H, *J* = 8.3, 2.6 Hz, 4-ArH), 5.39 (s, 2H, ArCH_2_), 4.41 (t, 2H, *J* = 6.7 Hz, CH_2_NO_3_), 3.94 (t, 2H, *J* = 6.3 Hz, OCH_2_), 2.54 (s, 3H, ArCH_3_), 2.47 (d, 6H, *J* = 8.9 Hz, ArCH_3_), 1.79–1.67 (m, 4H, CH_2_CH_2_CH_2_CH_2_CH_2_CH_2_), 1.51–1.39 (m, 4H, CH_2_CH_2_CH_2_CH_2_CH_2_CH_2_); ^13^C NMR (151 MHz, CDCl_3_) δ 166.11, 158.96, 151.40, 149.33, 148.97, 144.83, 130.99, 129.40, 122.07, 120.00, 114.84, 77.21, 77.00, 76.79, 73.16, 67.81, 65.91, 28.92, 26.69, 25.62, 25.42, 21.65, 21.40, 20.58; HRMS (ESI): m/z: 418.1978 calc. for C_21_H_28_N_3_O_6_ [M + H]^+^, found 418.1978, ppm error 0.0.

#### 3.1.15. Data Analysis of NMR Spectra

NMR spectra were analyzed using the software MestReNova 14.0 after confirming that the measured molecular mass in the HRMS of individual compounds matched their calculated value. The ^1^H NMR spectral peak reports of the compounds were exported after processing for peak picking, integration and multiplets analysis using the software MestReNova 14.0. Individual peaks’ data were analyzed, and the corresponding hydrogen atom was found in the compound structure. The peaks in ^13^C NMR spectra were also picked and reported using this software, and the data of the characteristic carbon peaks (C=O, – CH_3_) were analyzed.

### 3.2. In Vitro Antiplatelet Aggregation Assay Induced by ADP and AA

New Zealand white (NZW) rabbits were purchased from the Qinglongshan Animal Breeding Center. Our experimental protocols were approved by the Wuhan University of Science and Technology Institutional Animal Care and Use Committee (IACUC; 202033) and carried out in accordance with the EU Directive 2010/63/EU for animal experiments. Blood samples were withdrawn from the rabbit carotid artery and infused into a centrifuge tube containing 1/10 of the volume of 3.8% sodium citrate solution, followed by mixing. After centrifugation at 112× *g* for 15 min at room temperature to obtain the supernatants as platelet-rich plasma (PRP), the remaining blood was further centrifuged at 1008× *g* for another 15 min to obtain platelet-poor plasma (PPP). The PPP was used to adjust the PRP concentration to 1 × 10^8^ mL^−1^. The aggregation rate of the individual compounds was measured by Born’s turbidimetric method at 37 °C using a Platelet Aggregometer (LBY-NJ4A Platelet-Aggregometer, Beijing Precil Instrument Co., Beijing).

Briefly, the PRP (260 μL) was preincubated in duplicate for 5 min at 37 °C with the vehicle, individual compounds or reference drugs (TMP) at the same concentrations (0.1, 0.2, 0.4, 0.8, 1.6, 3.2 mM) and the platelet aggregation in the individual PPR samples was induced by adding 30 μL of ADP (final concentration of 10 μM) or AA (final concentration of 1 mM), followed by the recording of light transmission at maximal aggregation within 5 min. The rabbit PRP treated with vehicles was used as a positive control. The inhibition rate of the tested individual compounds was calculated as the following formula: inhibition rate (%) = [(1 − (the platelet aggregation in samples with the tested compound)/(the platelet aggregation in control samples)] × 100. The IC50 values of the tested compounds were determined by nonlinear regression analysis using GraphPad Prism 9.0. Data are expressed as the mean ± SD of each group (n = 5) and are analyzed by one-way analysis of variance (ANOVA) with Tukey’s test.

### 3.3. Molecular Docking

Molecular docking calculations were performed by Discovery Studio software using the TBXA2R. First, we downloaded the protein’s 3D structure PDB format file through the RSCB PDB database (PDB ID: 6IIV) and used hydrogenation and dehydration of the Macromolecules > Prepare Protein module in Discovery Studio software. Further, 6IIV is defined as a receptor through the Receptor-Ligand Interactions module. Set (X = 24.749525, Y = 162.147302, Z = 144.741603, Radius = 10.5000) as the active docking site [21]. The Chemoffice software was used to draw the structures of active compounds, and after importing them into the Discovery Studio software, the Small Molecules > Prepare or Filter Ligands module was used to prepare the compounds. After the preparation of the receptors and ligands, precise molecular docking between compounds and proteins was completed through the Receptor-Ligand Interactions > Dock Ligands (CDOCKER) module [22]. Further, -CDOCKER_INTERACTION_ENERGY TOP#1 was used as the screening condition to obtain the best results of the interaction between each compound and protein.

## 4. Conclusions

In conclusion, we designed and synthesized a series of novel nitric oxide-donating ligustrazine derivatives. All the derivatives exhibited inhibitory activities on platelet aggregation induced by ADP or AA in vitro. Compounds **14a** and **15d** displayed significantly better activity than ligustrazine in both assays. Preliminary in vitro studies have shown that it was a viable strategy for improving the effects due to the addition of the NO donor in the structure. Additionally, we studied docking with the novel derivatives at the active site of TBXA2R and discussed the structure-activity relationship, which will provide a reference for the research of this class of compounds. As a result, our present studies suggested that the novel NO-donating ligustrazine derivatives **14a** and **15d** deserve further study as potent antiplatelet aggregation agents.

## Figures and Tables

**Table 1:** Chemical structures of compounds **14a**–**14e** and **15a**–**15e**.

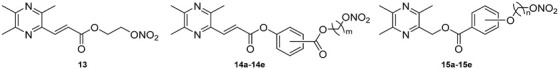		
Compounds	Substituted Position	Linker
**13**	/	/
**14a**	o-	m = 2
**14b**	m-	m = 2
**14c**	p-	m = 2
**14d**	o-	m = 4
**14e**	o-	m = 6
**15a**	o-	n = 2
**15b**	m-	n = 2
**15c**	p-	n = 2
**15d**	m-	n = 4
**15e**	m-	n = 6

## Data Availability

All data used to support the findings of this study are included within the article and Supplementary Materials.

## Supplementary Materials

The following supporting information can be downloaded at: https://www.mdpi.com/article/10.3390/molecules28083355/s1, Table S1: The IC_50_ values of target compounds against platelet aggregation in vitro induced by ADP or AA, Table S2: Docking energies of ligustrazine (TMP) and all new compounds (**13**, **14a**–**14e**, **15a**–**15e**) with TBXA2R. Binding modes of all the compounds at the active site of TBXA2R. ^1^H-NMR, ^13^C-NMR and HRMS spectroscopy of all new compounds.
