# Supplementary material for: Synthesis and Evaluation of Novel Nitric Oxide-Donating Ligustrazine Derivatives as Potent Antiplatelet Aggregation Agents
Source: Molecules. 2023 Apr 11;28(8):3355. doi: 10.3390/molecules28083355 (PMC10144142; doi:10.3390/molecules28083355)
Supplement: Supplementary file 1 [file molecules-28-03355-s001.zip › molecules-2300057-supplementary.pdf]

Supplementary Materials

Synthesis and evaluation of novel nitric oxide-donating ligustrazine derivatives as potent antiplatelet aggregation agents

Han-Xu Li, Jian-Hui Tian, Hua-Yu Li, Xin Wan and Yu Zou*
Institute of Pharmaceutical Process, Hubei Province Key Laboratory of Occupationl Hazard Identification and Control, School of Medicine, Wuhan University of Science and Technology, Wuhan 430065, China
*Correspondence: zouyu@wust.edu.cn

1 Table S1: The IC_{50} values of target compounds against platelet aggregation in vitro induced by ADP or AA……………………………………………………S4
2 Table S2: Docking energies of ligustrazine (TMP) and all new compounds (**13**, **14a**-**14e**, **15a**-**15e**) with TBXA2R………………………………………………S4
3 Binding modes of all the compounds at active site of TBXA2R
3.1 Figure S1. Possible binding mode of TMP at active site of TBXA2R……S5
3.2 Figure S2. Possible binding mode of **13** at active site of TBXA2R………S6
3.3 Figure S3. Possible binding mode of **14a** at active site of TBXA2R………S7
3.4 Figure S4. Possible binding mode of **14b** at active site of TBXA2R………S8
3.5 Figure S5. Possible binding mode of **14c** at active site of TBXA2R………S9
3.6 Figure S6. Possible binding mode of **14d** at active site of TBXA2R…….S10
3.7 Figure S7. Possible binding mode of **14e** at active site of TBXA2R…….S11
3.8 Figure S8. Possible binding mode of **15a** at active site of TBXA2R……S12
3.9 Figure S9. Possible binding mode of **15b** at active site of TBXA2R……S13
3.10 Figure S10. Possible binding mode of **15c** at active site of TBXA2R……S14
3.11 Figure S11. Possible binding mode of **15d** at active site of TBXA2R……S15
3.12 Figure S12. Possible binding mode of **15e** at active site of TBXA2R……S16
4 NMR spectra of all new compounds
4.1 Figure S13. ^{1}H NMR spectrum of 2-(nitrooxy)ethyl (E)-3-(3,5,6-trimethylpyrazin-2-yl)acrylate (**13**)……………………………………..S17
4.2 Figure S14. ^{1}H NMR spectrum of 2-(nitrooxy)ethyl (E)-2-((3-(3,5,6-trimethylpyrazin-2-yl)acryloyl)oxy)benzoate (**14a**)…..............S17
4.3 Figure S15. ^{1}H NMR spectrum of 2-(nitrooxy)ethyl (E)-3-((3-(3,5,6-trimethylpyrazin-2-yl)acryloyl)oxy)benzoate (**14b**)………….S18
4.4 Figure S16. ^{1}H NMR spectrum of 2-(nitrooxy)ethyl (E)-4-((3-(3,5,6-trimethylpyrazin-2-yl)acryloyl)oxy)benzoate (**14c**)…………..S18
4.5 Figure S17. ^{1}H NMR spectrum of 4-(nitrooxy)butyl (E)-2-((3-(3,5,6-trimethylpyrazin-2-yl)acryloyl)oxy)benzoate (**14d**)………….S19
4.6 Figure S18. ^{1}H NMR spectrum of 6-(nitrooxy)hexyl (E)-2-((3-(3,5,6-trimethylpyrazin-2-yl)acryloyl)oxy)benzoate (**14e**)…………..S19

4.7 Figure S19. ^{1}H NMR spectrum of (3,5,6-trimethylpyrazin-2-yl)methyl 2-(2-(nitrooxy)ethoxy)benzoate (**15a**)…………………………………...S20
4.8 Figure S20. ^{1}H NMR spectrum of (3,5,6-trimethylpyrazin-2-yl)methyl 3-(2-(nitrooxy)ethoxy)benzoate (**15b**)…………………………………...S20
4.9 Figure S21. ^{1}H NMR spectrum of (3,5,6-trimethylpyrazin-2-yl)methyl 4-(2-(nitrooxy)ethoxy)benzoate (**15c**)…………………………………...S21
4.10 Figure S22. ^{1}H NMR spectrum of (3,5,6-trimethylpyrazin-2-yl)methyl 3-(4-(nitrooxy)butoxy)benzoate (**15d**)…………………………………...S21
4.11 Figure S23. ^{1}H NMR spectrum of (3,5,6-trimethylpyrazin-2-yl)methyl 3-((6-(nitrooxy)hexyl)oxy)benzoate (**15e**)…………………………….S22
4.12 Figure S24. ^{13}C NMR spectrum of 2-(nitrooxy)ethyl (E)-3-(3,5,6-trimethylpyrazin-2-yl)acrylate (**13**)…………………………….S22
4.13 Figure S25. ^{13}C NMR spectrum of 2-(nitrooxy)ethyl (E)-2-((3-(3,5,6-trimethylpyrazin-2-yl)acryloyl)oxy)benzoate (**14a**)………….S23
4.14 Figure S26. ^{13}C NMR spectrum of 2-(nitrooxy)ethyl (E)-3-((3-(3,5,6-trimethylpyrazin-2-yl)acryloyl)oxy)benzoate (**14b**)………….S23
4.15 Figure S27. ^{13}C NMR spectrum of 2-(nitrooxy)ethyl (E)-4-((3-(3,5,6-trimethylpyrazin-2-yl)acryloyl)oxy)benzoate (**14c**)…………..S24
4.16 Figure S28. ^{13}C NMR spectrum of 4-(nitrooxy)butyl (E)-2-((3-(3,5,6-trimethylpyrazin-2-yl)acryloyl)oxy)benzoate (**14d**…………..S24
4.17 Figure S29. ^{13}C NMR spectrum of 6-(nitrooxy)hexyl (E)-2-((3-(3,5,6-trimethylpyrazin-2-yl)acryloyl)oxy)benzoate (**14e**)…………..S25
4.18 Figure S30. ^{13}C NMR spectrum of (3,5,6-trimethylpyrazin-2-yl)methyl 2-(2-(nitrooxy)ethoxy)benzoate (**15a**)…………………………………...S25
4.19 Figure S31. ^{13}C NMR spectrum of (3,5,6-trimethylpyrazin-2-yl)methyl 3-(2-(nitrooxy)ethoxy)benzoate (**15b**)…………………………………...S26
4.20 Figure S32. ^{13}C NMR spectrum of (3,5,6-trimethylpyrazin-2-yl)methyl 4-(2-(nitrooxy)ethoxy)benzoate (**15c**)…………………………………...S26
4.21 Figure S33. ^{13}C NMR spectrum of (3,5,6-trimethylpyrazin-2-yl)methyl 3-(4-(nitrooxy)butoxy)benzoate (**15d**)…………………………………...S27
4.22 Figure S34. ^{13}C NMR spectrum of (3,5,6-trimethylpyrazin-2-yl)methyl 3-((6-(nitrooxy)hexyl)oxy)benzoate (**15e**)…………………………….S27
5 HRMS spectra of all new compounds
5.1 Figure S35. HRMS spectrum of 2-(nitrooxy)ethyl (E)-3-(3,5,6-trimethylpyrazin-2-yl)acrylate (**13**)…………………………….S28
5.2 Figure S36. HRMS spectrum of 2-(nitrooxy)ethyl (E)-2-((3-(3,5,6-trimethylpyrazin-2-yl)acryloyl)oxy)benzoate (**14a**)………….S28
5.3 Figure S37. HRMS spectrum of 2-(nitrooxy)ethyl (E)-3-((3-(3,5,6-trimethylpyrazin-2-yl)acryloyl)oxy)benzoate (**14b**)………….S29
5.4 Figure S38. HRMS spectrum of 2-(nitrooxy)ethyl (E)-4-((3-(3,5,6-trimethylpyrazin-2-yl)acryloyl)oxy)benzoate (**14c**)………….S29
5.5 Figure S39. HRMS spectrum of 4-(nitrooxy)butyl (E)-2-((3-(3,5,6-trimethylpyrazin-2-yl)acryloyl)oxy)benzoate (**14d**)………….S30
5.6 Figure S40. HRMS spectrum of 6-(nitrooxy)hexyl (E)-2-

((3-(3,5,6-trimethylpyrazin-2-yl)acryloyl)oxy)benzoate (**14e**)…………..S30
5.7 Figure S41. HRMS spectrum of (3,5,6-trimethylpyrazin-2-yl)methyl 2-(2-(nitrooxy)ethoxy)benzoate (**15a**)…………………………………...S31
5.8 Figure S42. HRMS spectrum of (3,5,6-trimethylpyrazin-2-yl)methyl 3-(2-(nitrooxy)ethoxy)benzoate (**15b**…………………………………....S31
5.9 Figure S43. HRMS spectrum of (3,5,6-trimethylpyrazin-2-yl)methyl 4-(2-(nitrooxy)ethoxy)benzoate (**15c**)…………………………………...S32
5.10 Figure S44. HRMS spectrum of (3,5,6-trimethylpyrazin-2-yl)methyl 3-(4-(nitrooxy)butoxy)benzoate (**15d**)…………………………………...S32
5.11 Figure S45. HRMS spectrum of (3,5,6-trimethylpyrazin-2-yl)methyl 3-((6-(nitrooxy)hexyl)oxy)benzoate (**15e**)……………………………….S33

Table S1. The IC_{50} values of target compounds against platelet aggregation in vitro induced by ADP or AA. (Mean ± SD, n = 5)

Compound	IC_{50} (mM)	
	ADP (10μM)	AA (1mM)
TMP	1.1100 ± 0.07	0.8155 ± 0.08
**13**	2.0810 ± 0.09	1.7120 ± 0.21
**14a**	0.7736 ± 0.08	0.6232 ± 0.05
**14b**	1.7830 ± 0.15	1.2390 ± 0.15
**14c**	2.2970 ± 0.20	1.8830 ± 0.14
**14d**	1.4380 ± 0.18	1.0400 ± 0.08
**14e**	2.5180 ± 0.26	2.3080 ± 0.30
**15a**	1.1710 ± 0.09	1.6270 ± 0.16
**15b**	0.7854 ± 0.06	0.8505 ± 0.03
**15c**	0.9230 ± 0.08	0.9658 ± 0.09
**15d**	0.7347 ± 0.07	0.5565 ± 0.03
**15e**	1.2060 ± 0.08	0.9412 ± 0.07

Table S2: Docking energies of ligustrazine (TMP) and all new compounds (**13**, **14a-14e**, **15a-15e**) with TBXA2R

Compound	-CDOCKER_INTERACTION_ENERGY, kcal/mol	-CDOCKER_ENERGY, kcal/mol
TMP	22.6805	15.7133
**13**	43.7139	26.4085
**14a**	57.2490	30.5841
**14b**	56.3094	38.3704
**14c**	57.3570	38.5336
**14d**	60.4573	28.8552
**14e**	56.2685	25.4620
**15a**	52.6835	27.3096
**15b**	54.9192	35.3326
**15c**	53.8605	29.0482
**15d**	61.9653	42.8464
**15e**	62.0654	38.7112

Binding modes of all the compounds at active site of TBXA2R

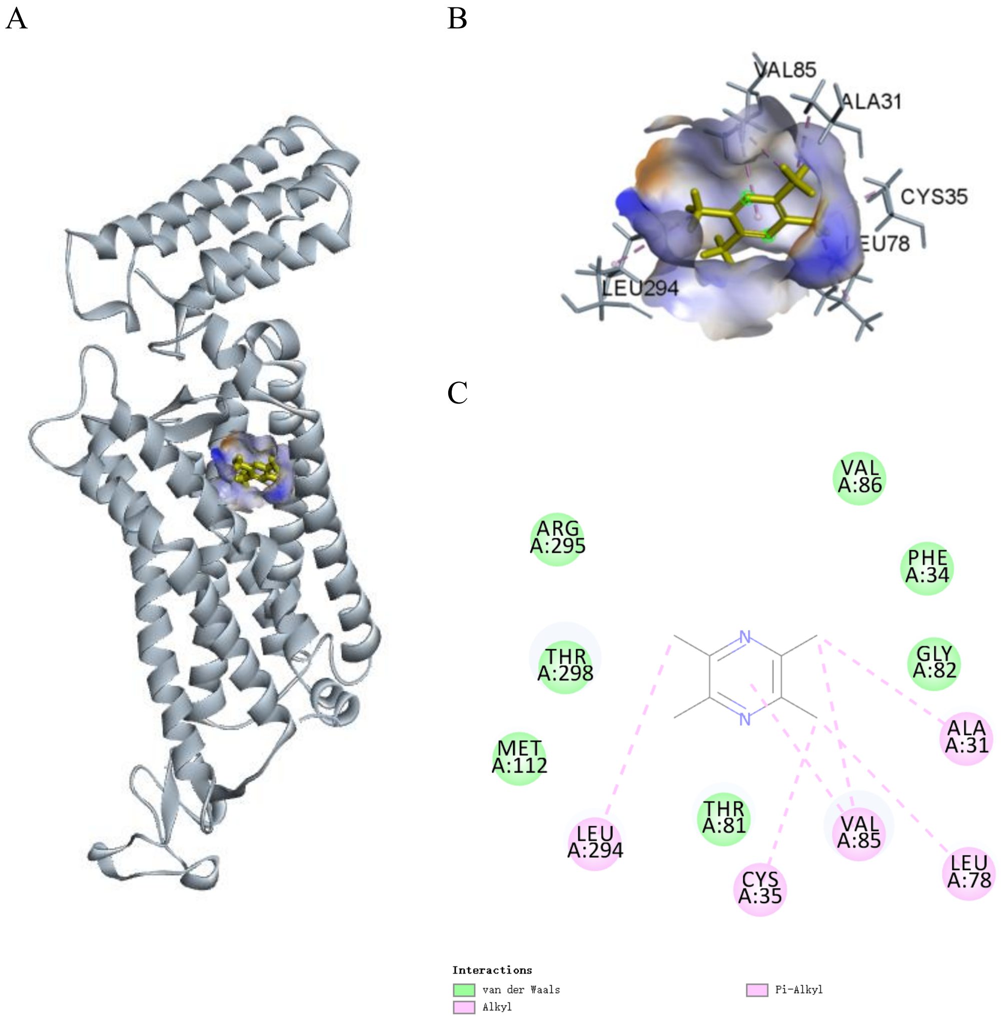

Figure S1. Possible binding mode of TMP at active site of TBXA2R.

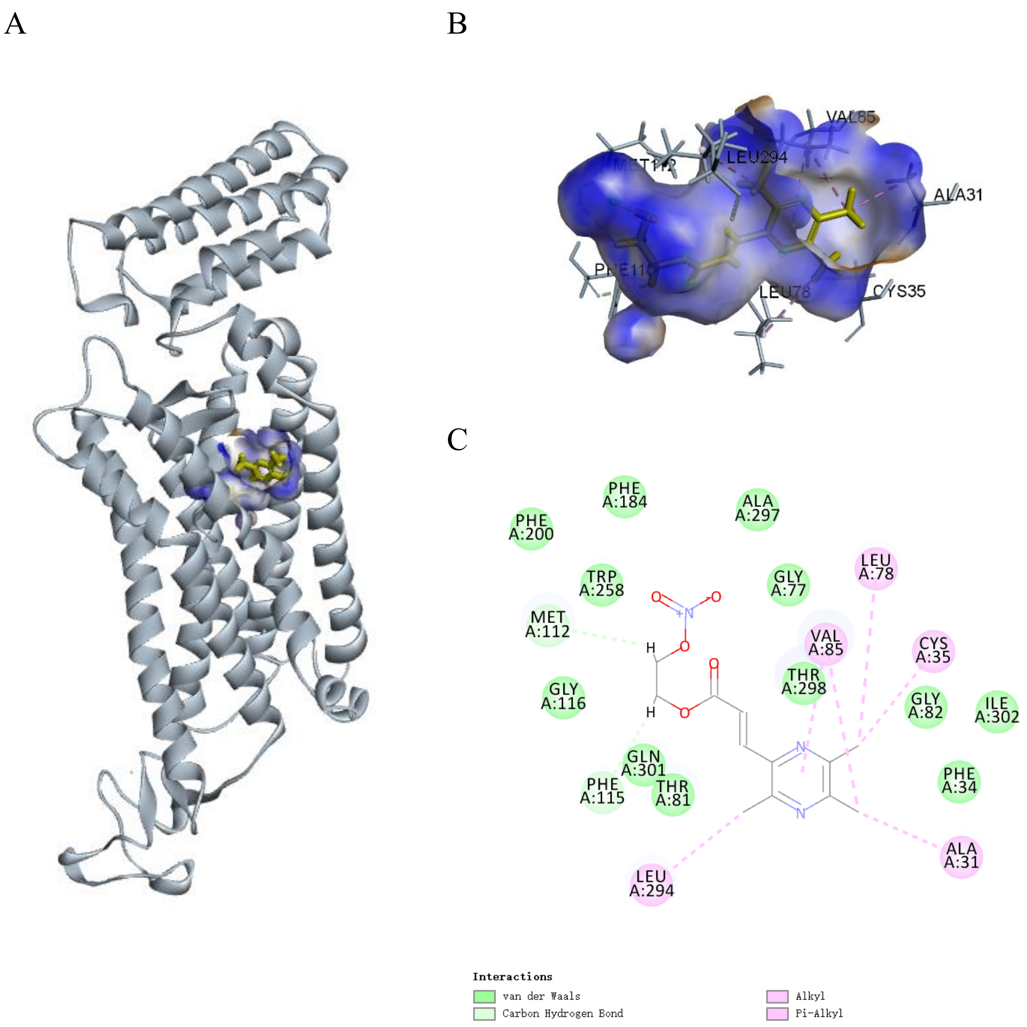

Figure S2. Possible binding mode of **13** at active site of TBXA2R.

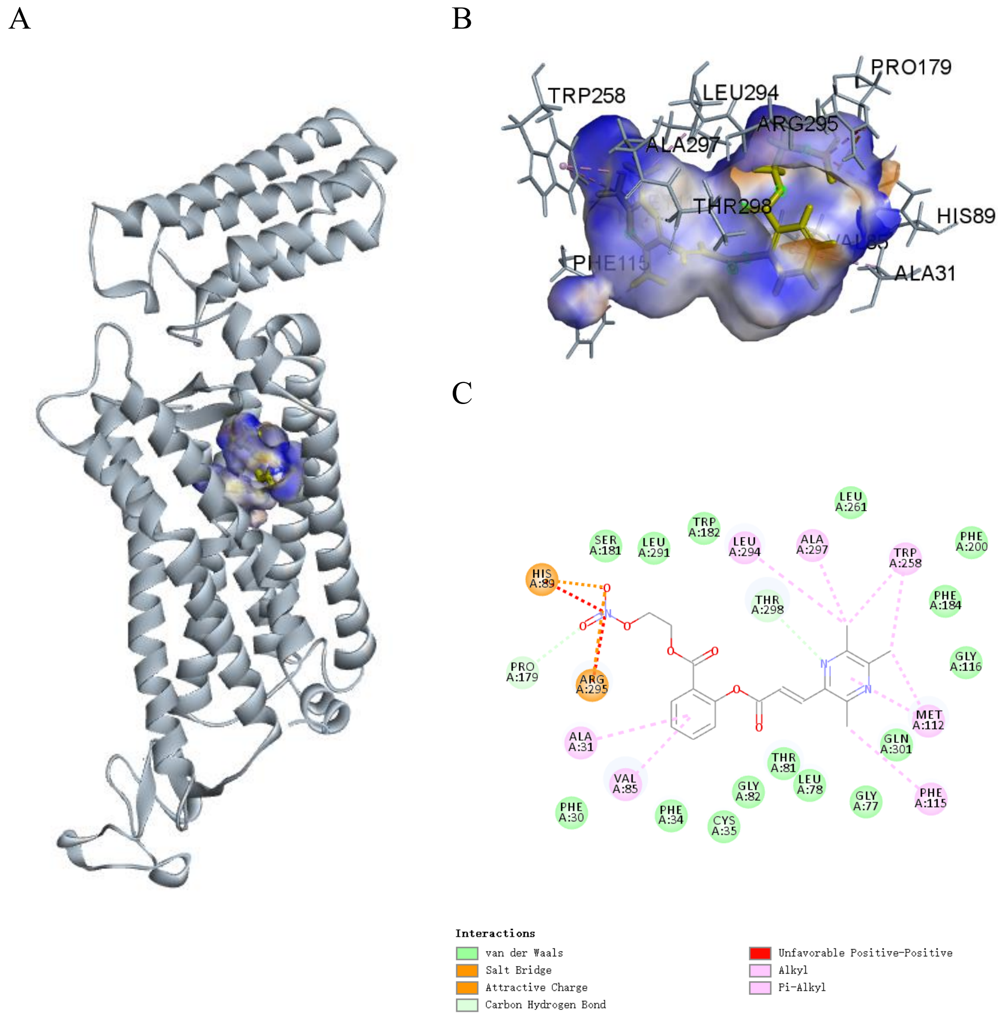

Figure S3. Possible binding mode of **14a** at active site of TBXA2R.

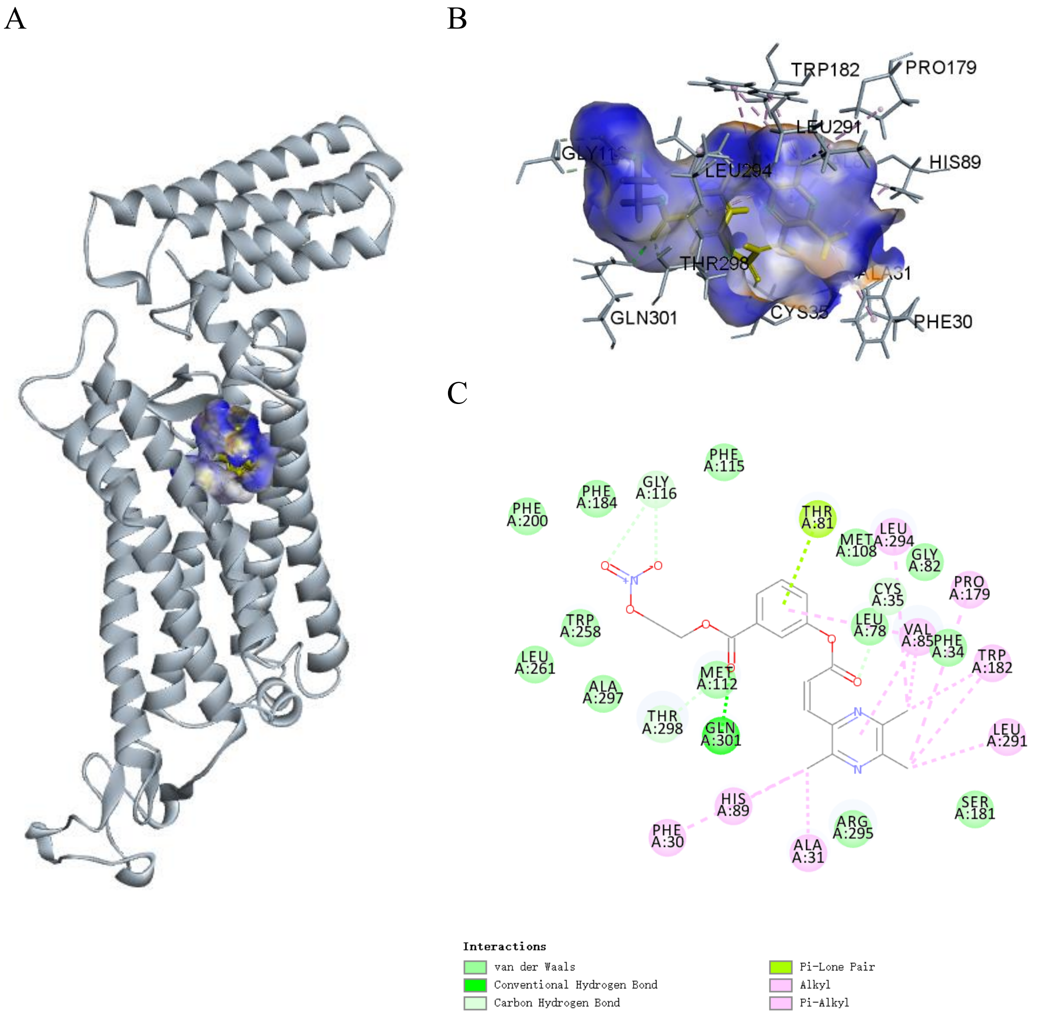

Figure S4. Possible binding mode of **14b** at active site of TBXA2R.

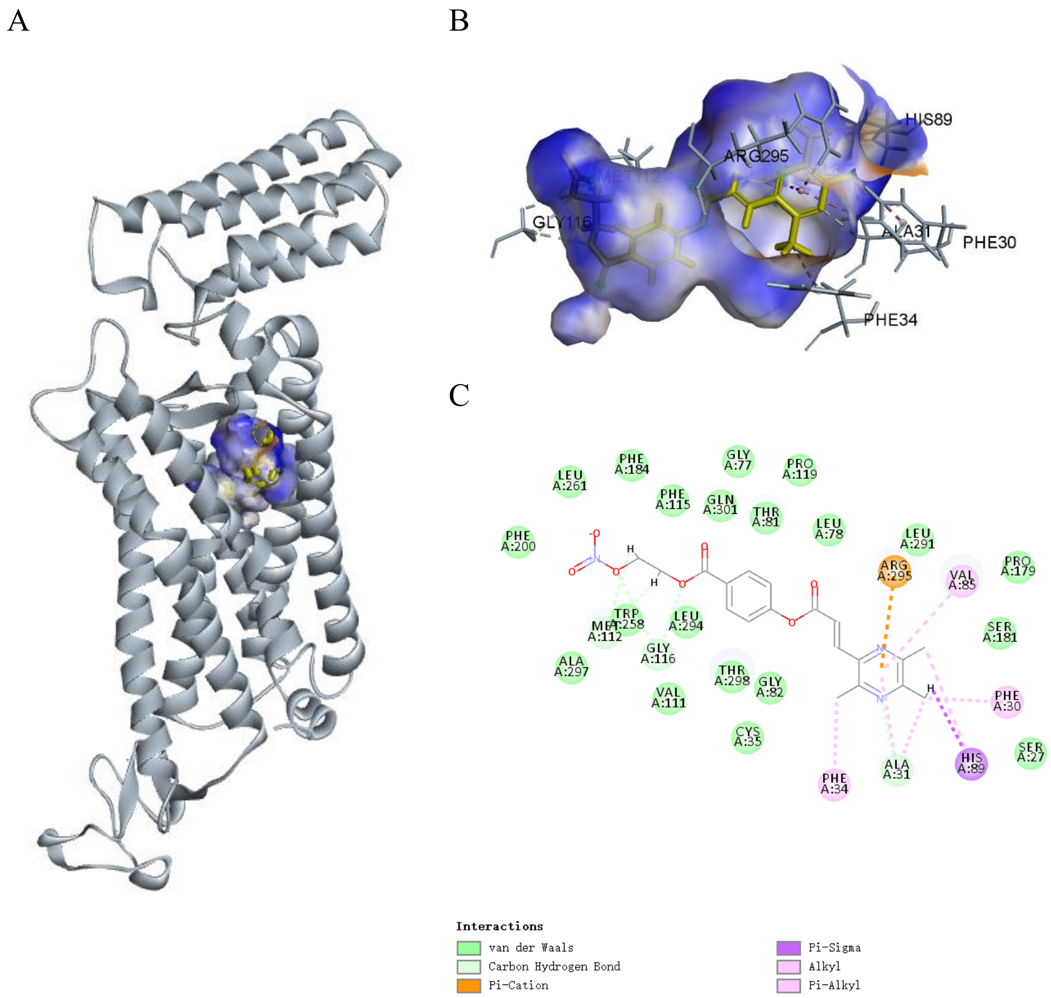

Figure S5. Possible binding mode of **14c** at active site of TBXA2R.

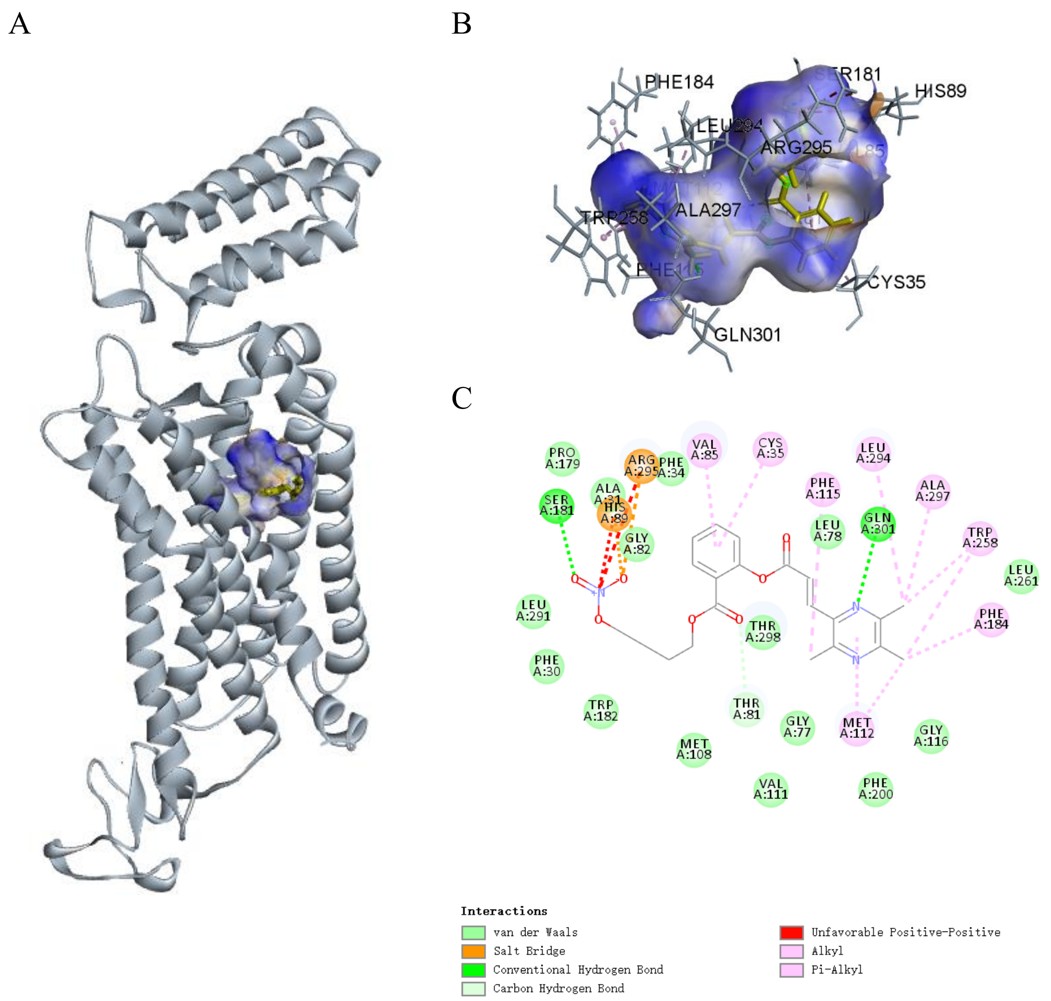

Figure S6. Possible binding mode of **14d** at active site of TBXA2R.

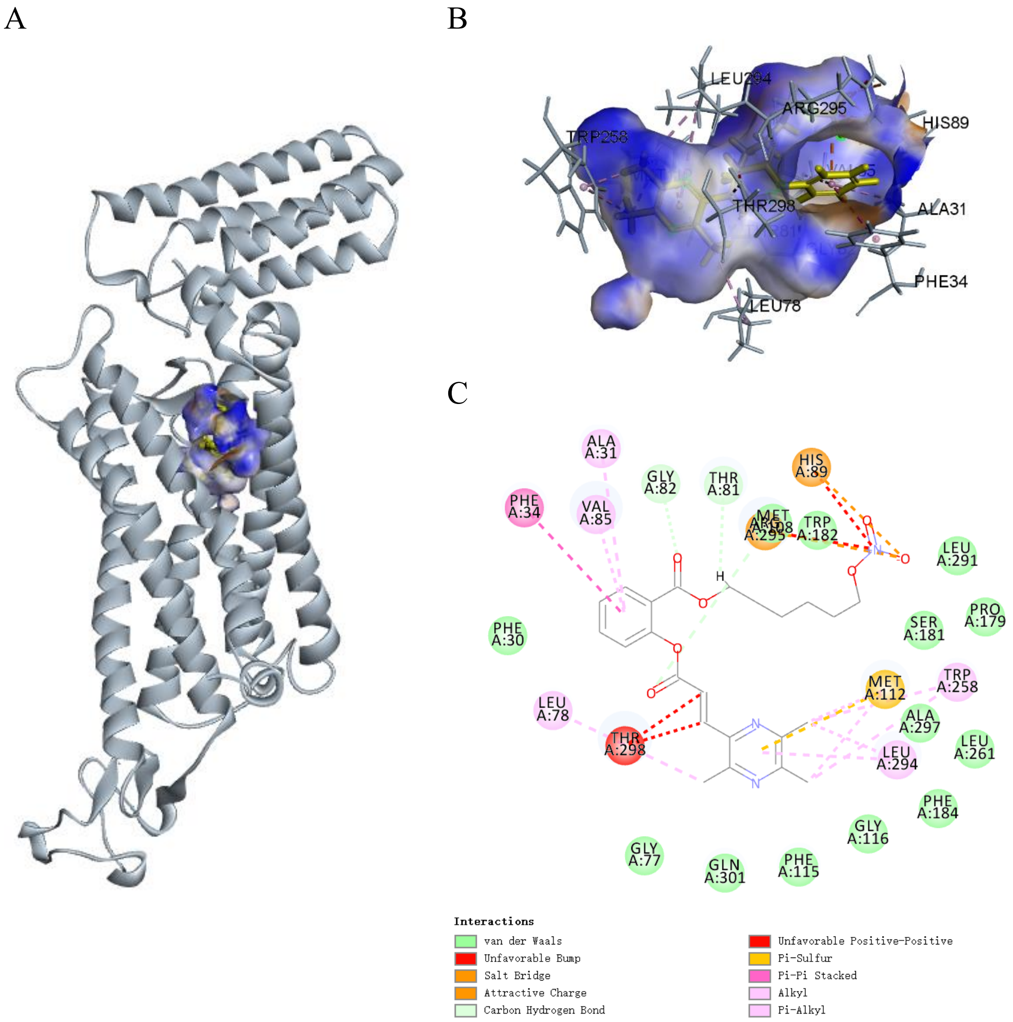

Figure S7. Possible binding mode of **14e** at active site of TBXA2R.

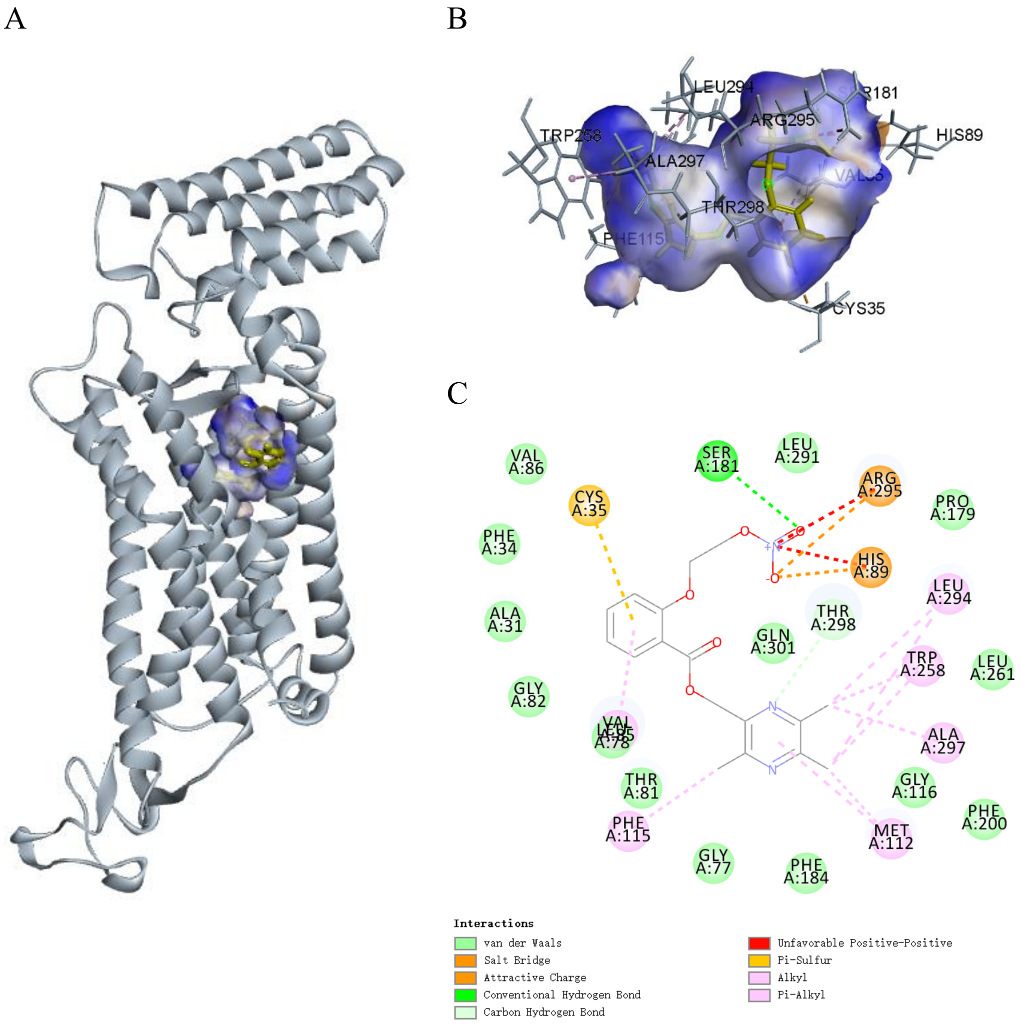

Figure S8. Possible binding mode of **15a** at active site of TBXA2R.

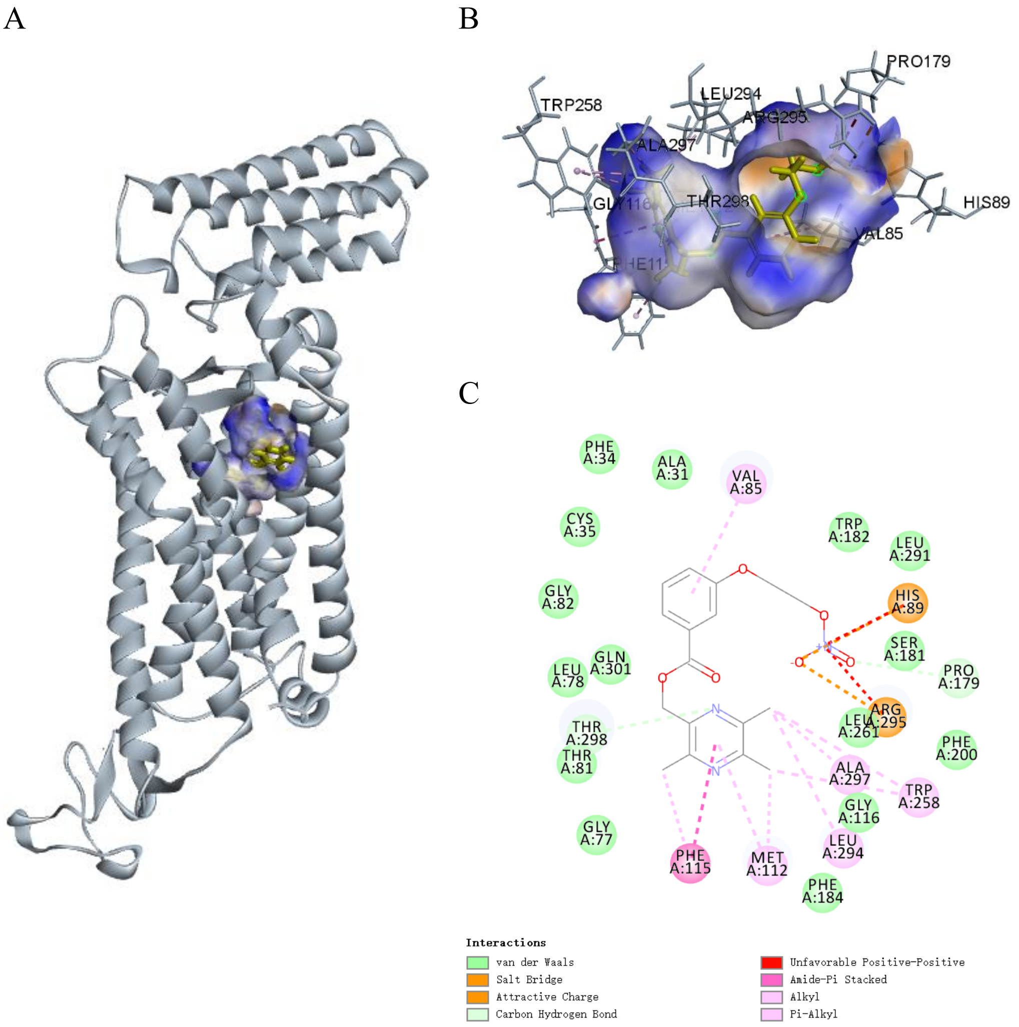

Figure S9. Possible binding mode of **15b** at active site of TBXA2R.

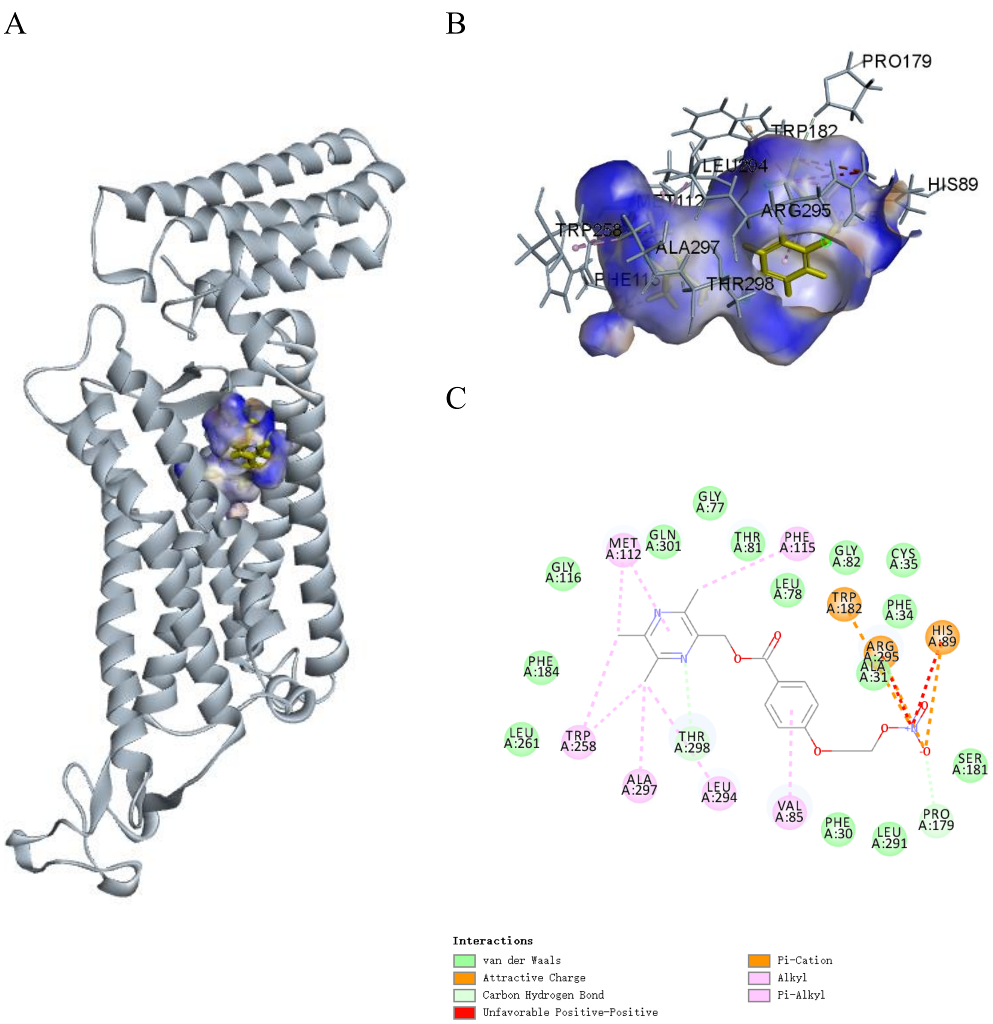

Figure S10. Possible binding mode of **15c** at active site of TBXA2R.

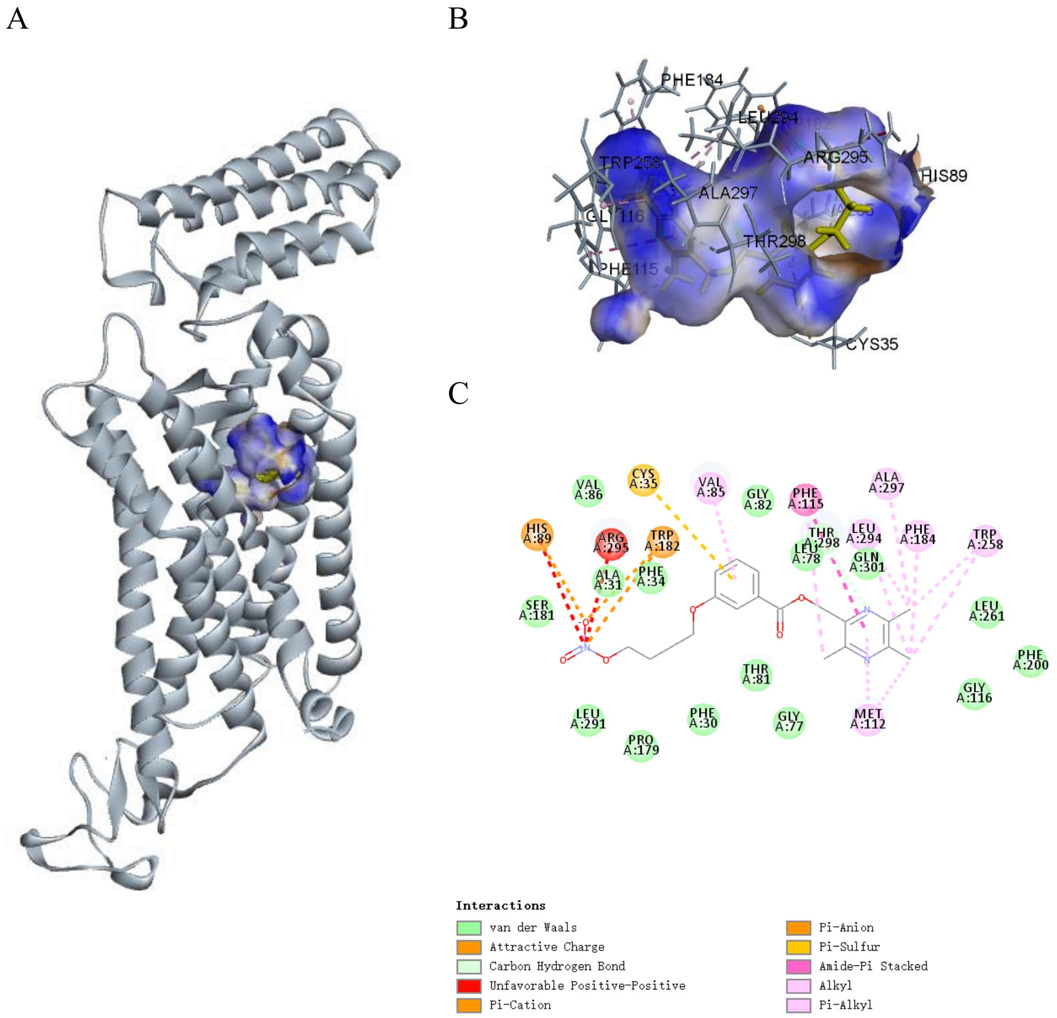

Figure S11. Possible binding mode of **15d** at active site of TBXA2R.

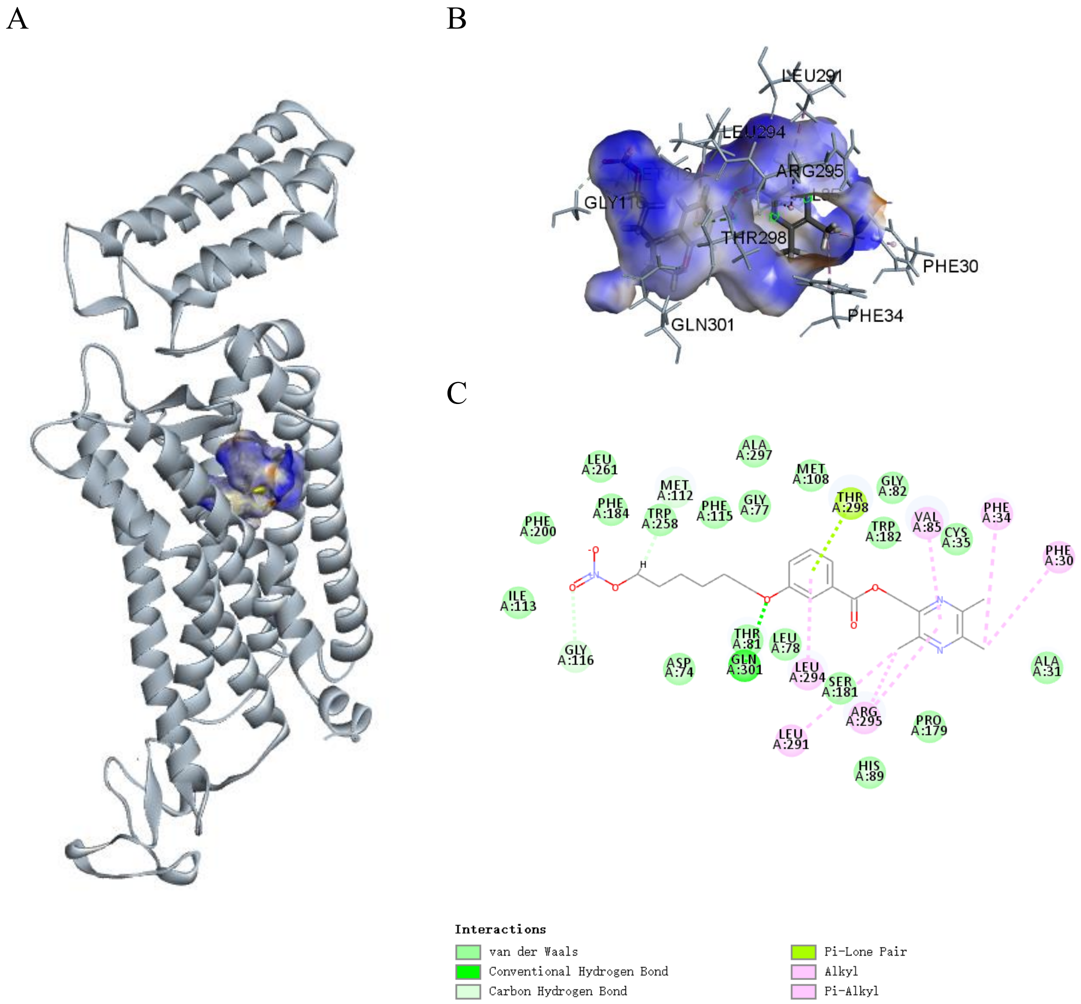

Figure S12. Possible binding mode of **15e** at active site of TBXA2R.

NMR spectra of all new compounds

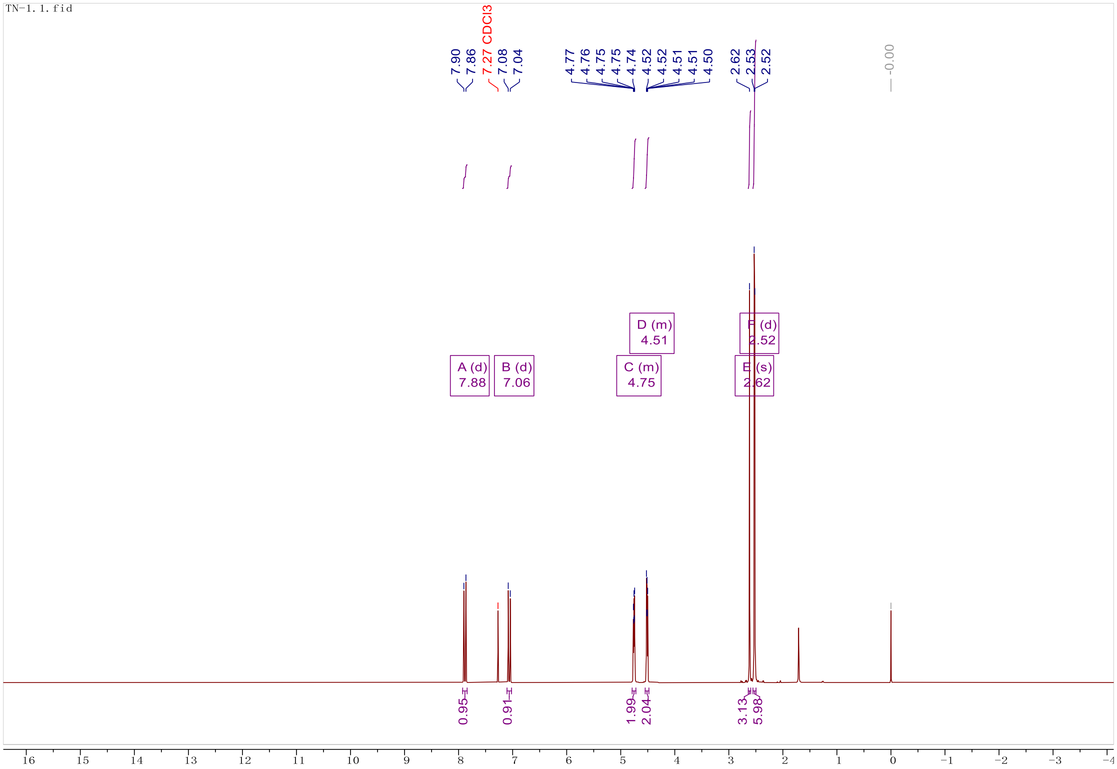

Figure S13. 1H NMR spectrum of 2-(nitrooxy)ethyl (E)-3-(3,5,6-trimethylpyrazin-2-yl)acrylate (**13**)

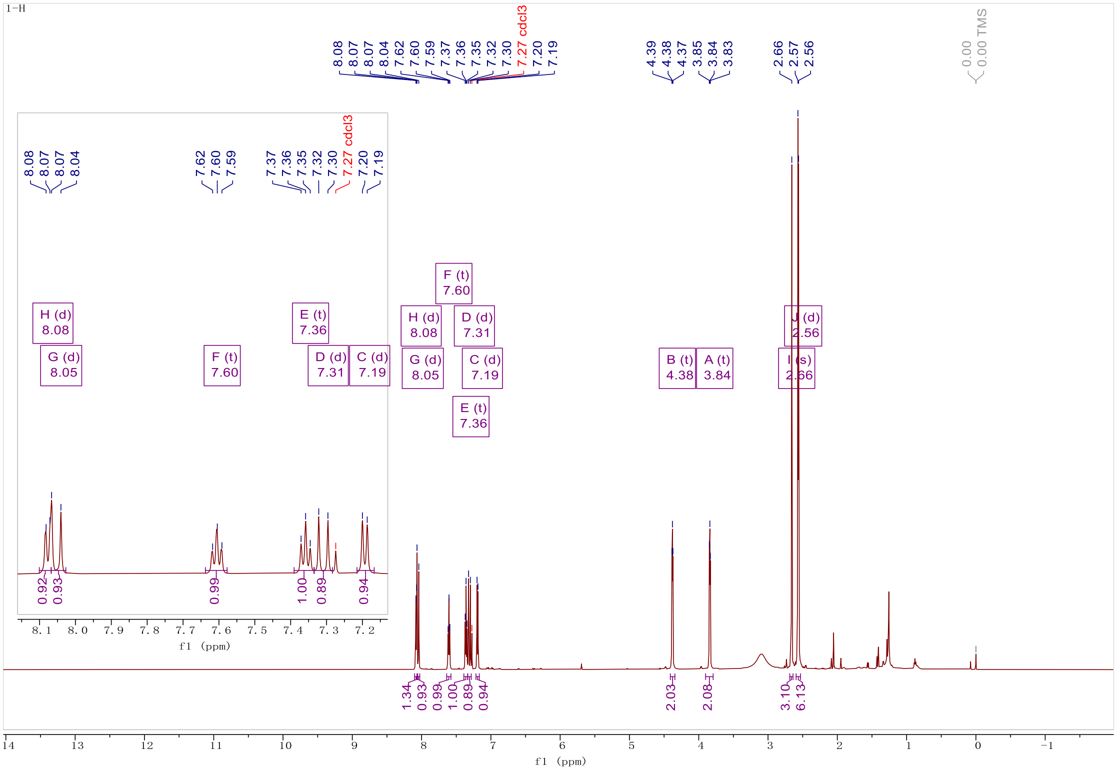

Figure S14. 1H NMR spectrum of 2-(nitrooxy)ethyl (E)-2-((3-(3,5,6-trimethylpyrazin-2-yl)acryloyl)oxy)benzoate (**14a**)

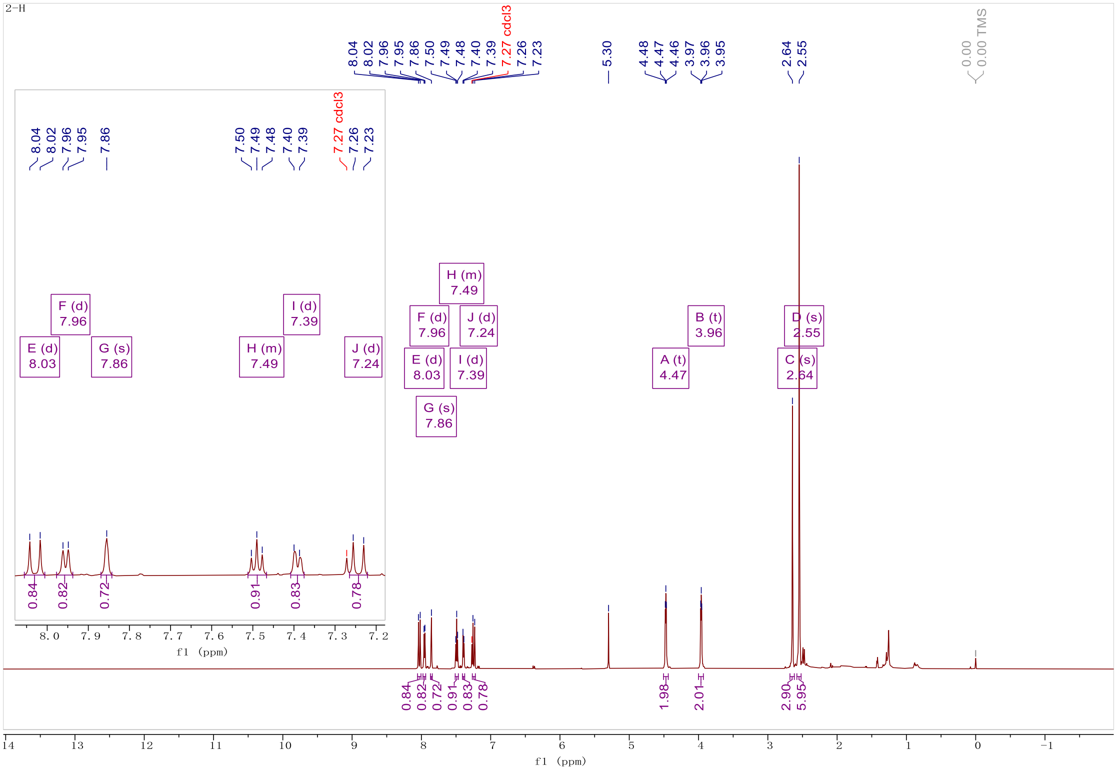

Figure S15. 1H NMR spectrum of 2-(nitrooxy)ethyl (E)-3-((3-(3,5,6-trimethylpyrazin-2-yl)acryloyl)oxy)benzoate (**14b**)

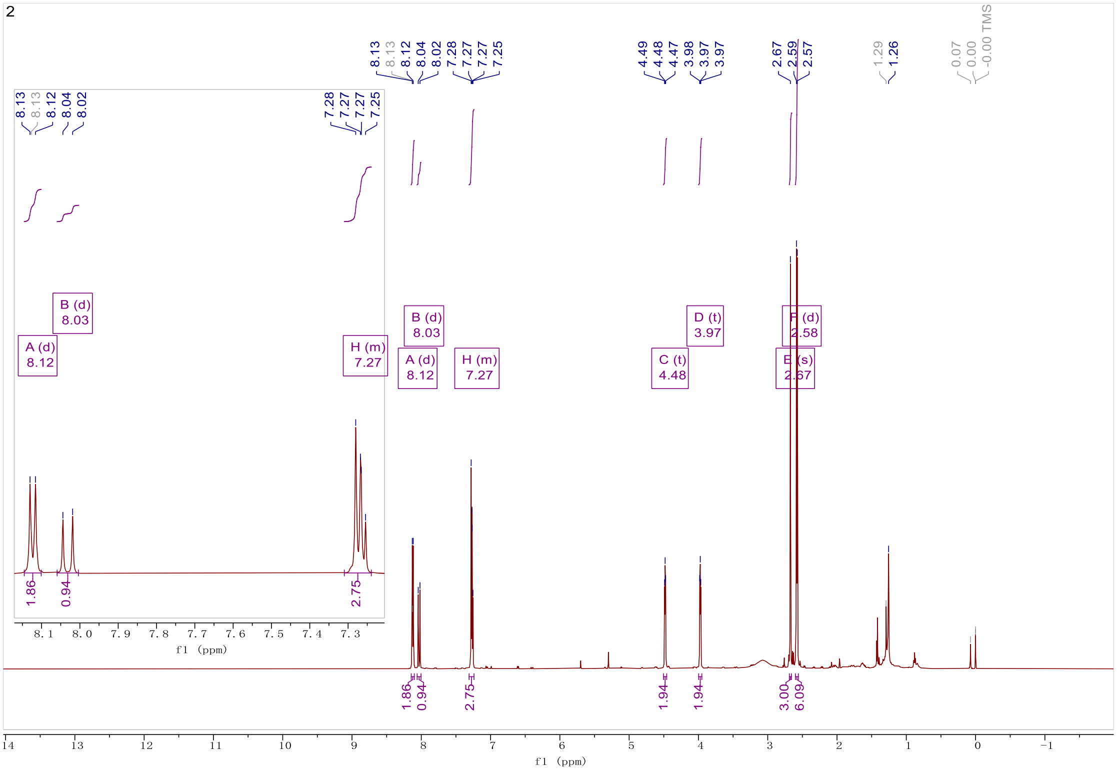

Figure S16. 1H NMR spectrum of 2-(nitrooxy)ethyl (E)-4-((3-(3,5,6-trimethylpyrazin-2-yl)acryloyl)oxy)benzoate (**14c**)

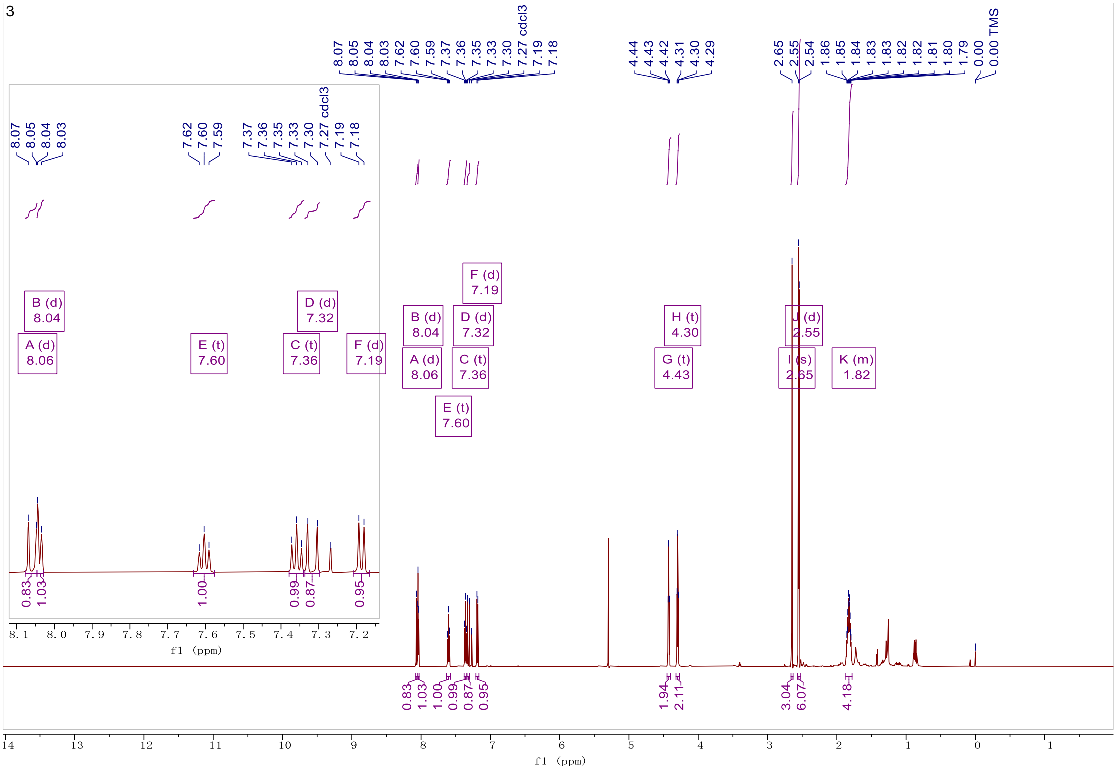

Figure S17. 1H NMR spectrum of 4-(nitrooxy)butyl (E)-2-((3-(3,5,6-trimethylpyrazin-2-yl)acryloyl)oxy)benzoate (**14d**)

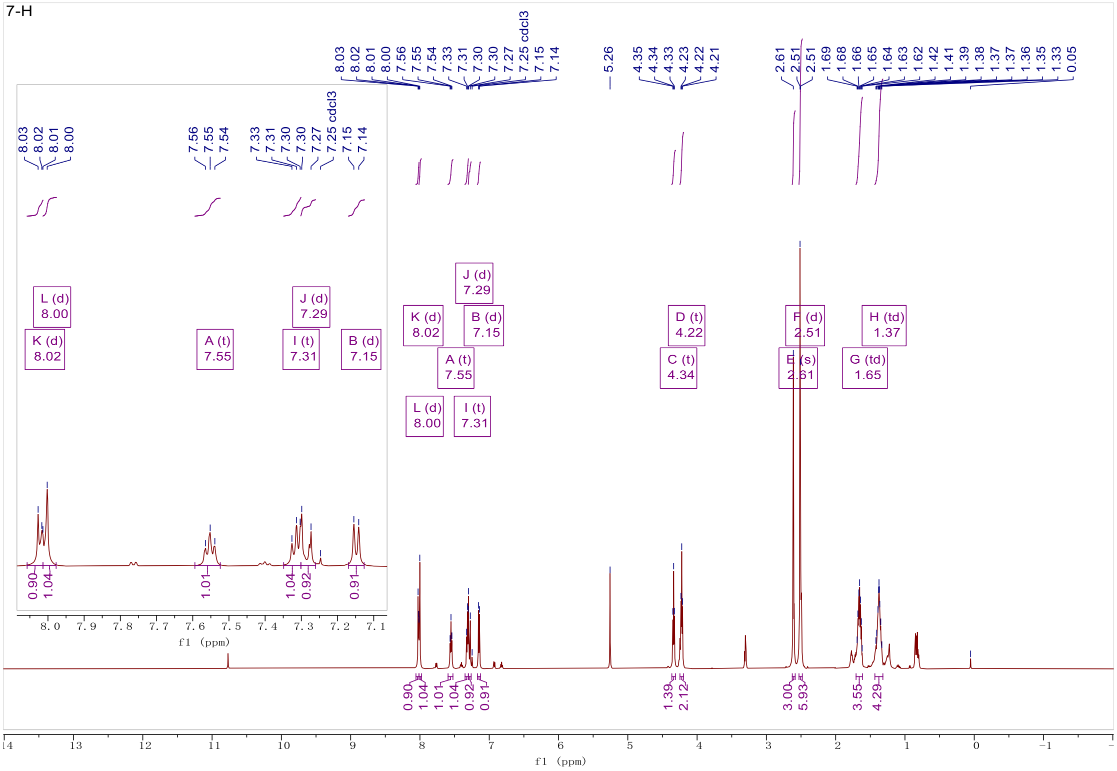

Figure S18. 1H NMR spectrum of 6-(nitrooxy)hexyl (E)-2-((3-(3,5,6-trimethylpyrazin-2-yl)acryloyl)oxy)benzoate (**14e**)

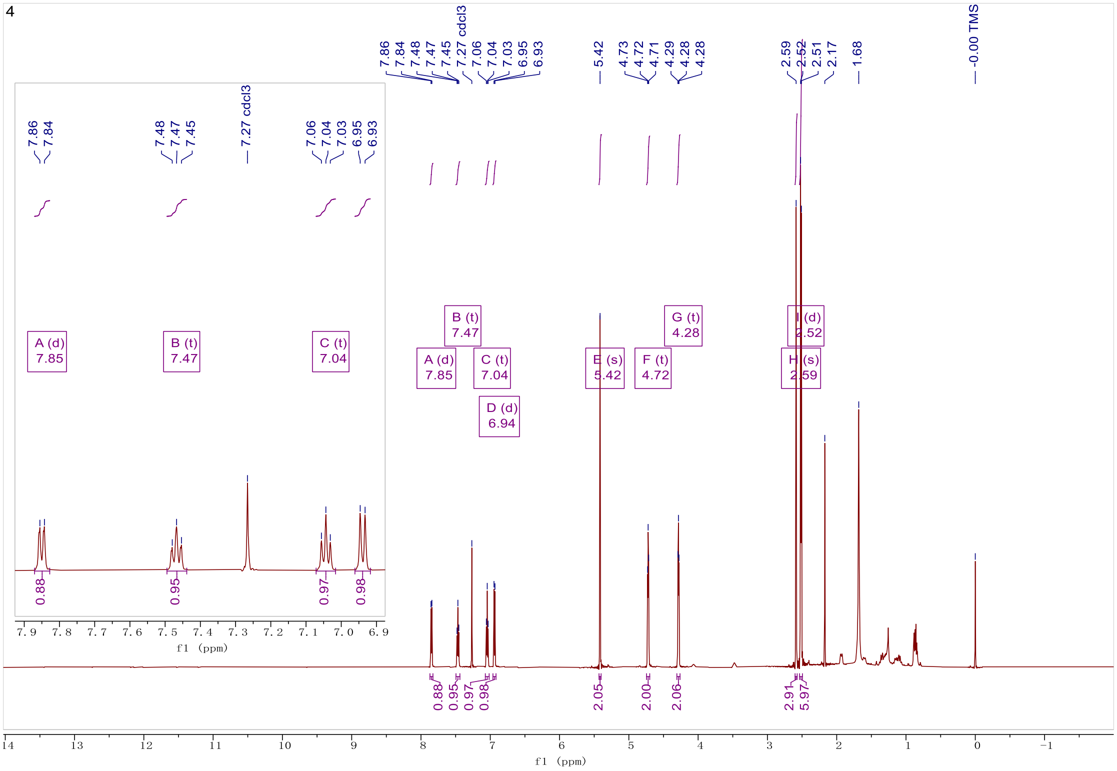

Figure S19. 1H NMR spectrum of (3,5,6-trimethylpyrazin-2-yl)methyl 2-(2-(nitrooxy)ethoxy)benzoate (**15a**)

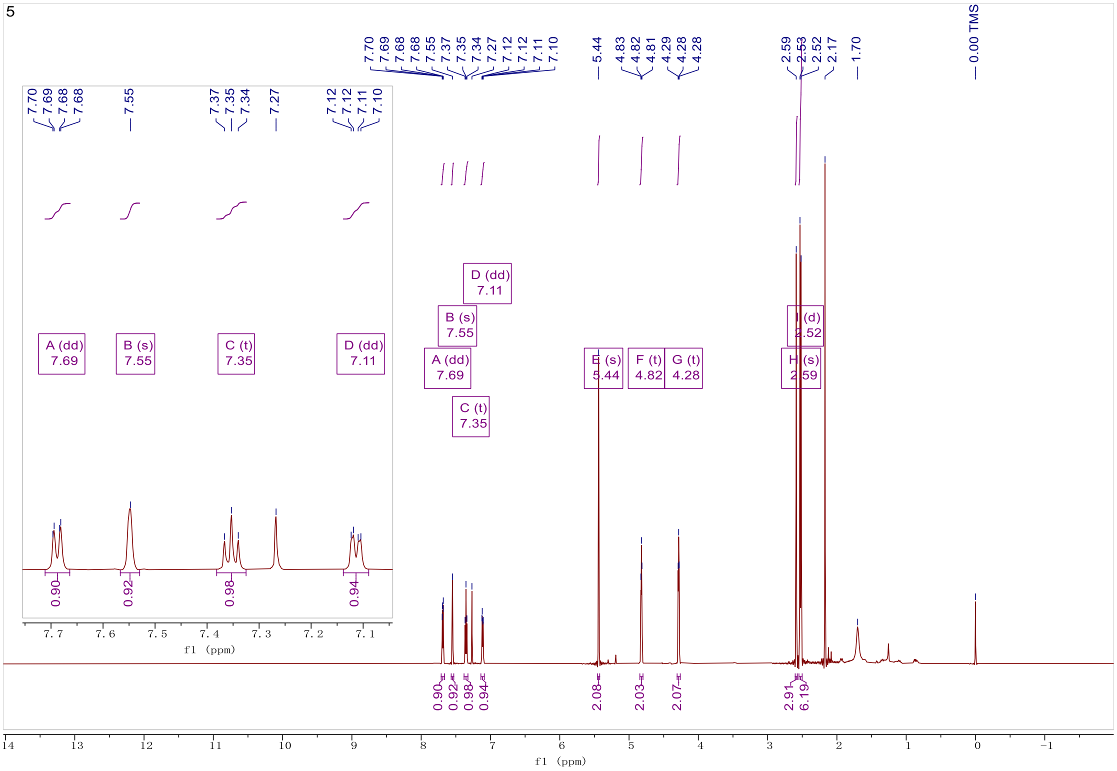

Figure S20. 1H NMR spectrum of (3,5,6-trimethylpyrazin-2-yl)methyl 3-(2-(nitrooxy)ethoxy)benzoate (**15b**)

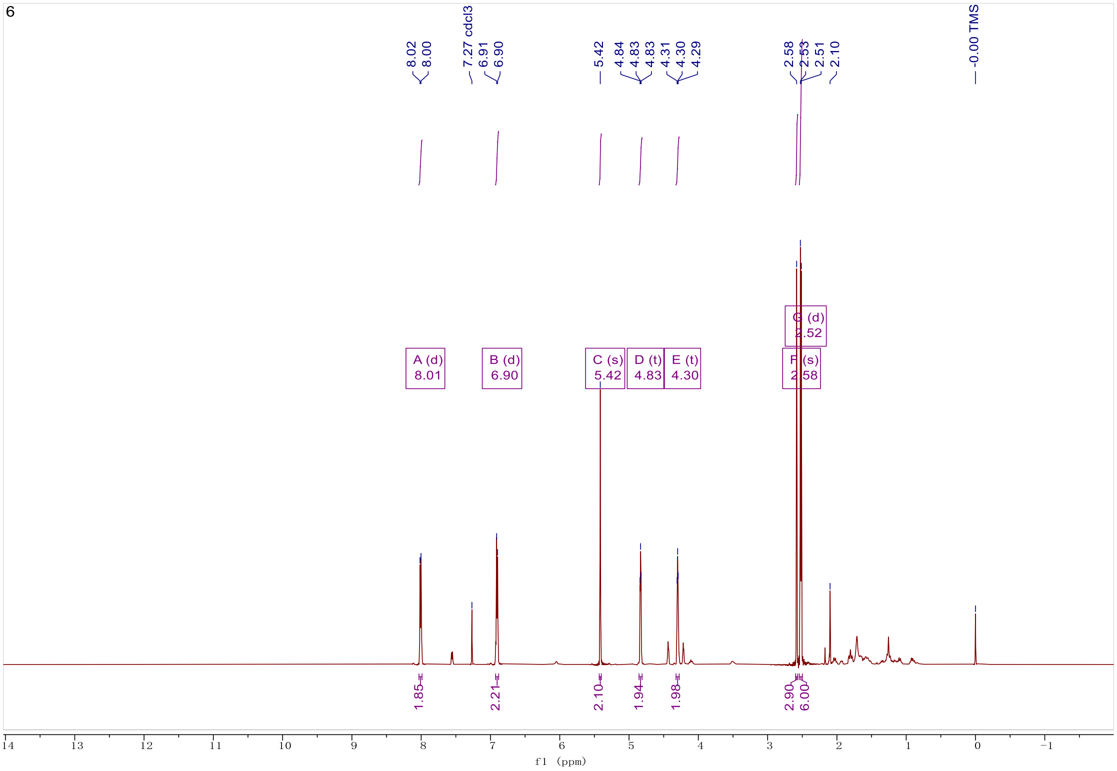

Figure S21. 1H NMR spectrum of (3,5,6-trimethylpyrazin-2-yl)methyl 4-(2-(nitrooxy)ethoxy)benzoate (**15c**)

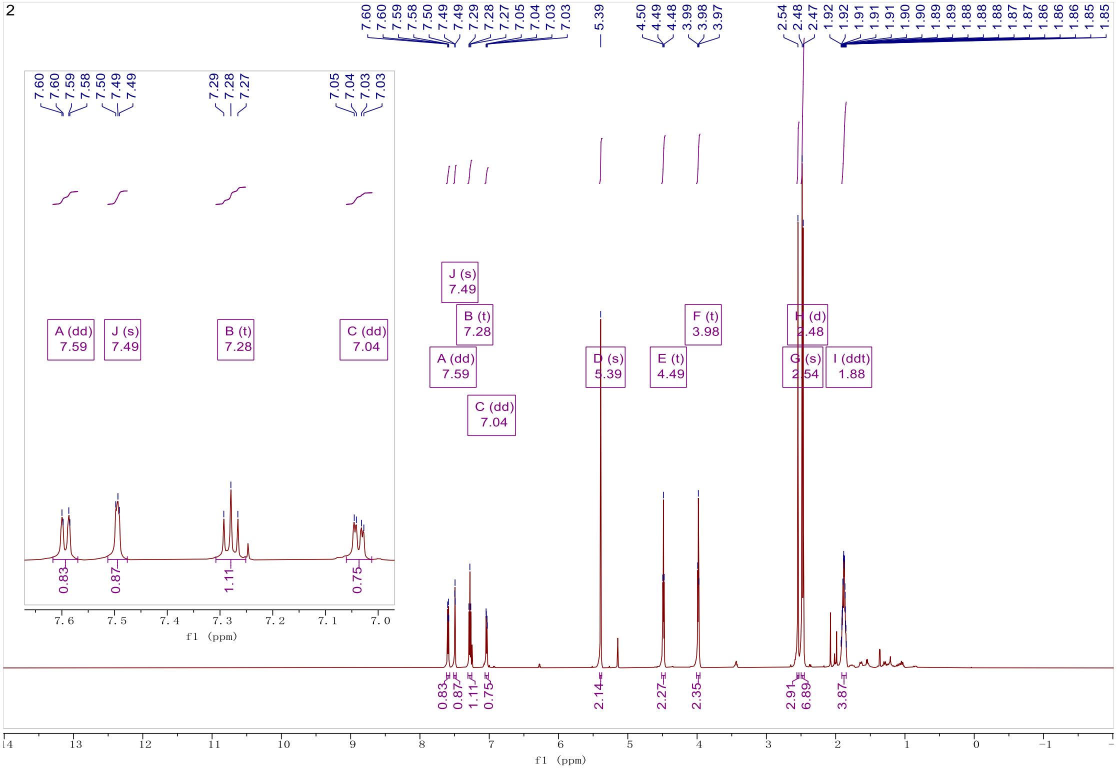

Figure S22. 1H NMR spectrum of (3,5,6-trimethylpyrazin-2-yl)methyl 3-(4-(nitrooxy)butoxy)benzoate (**15d**)

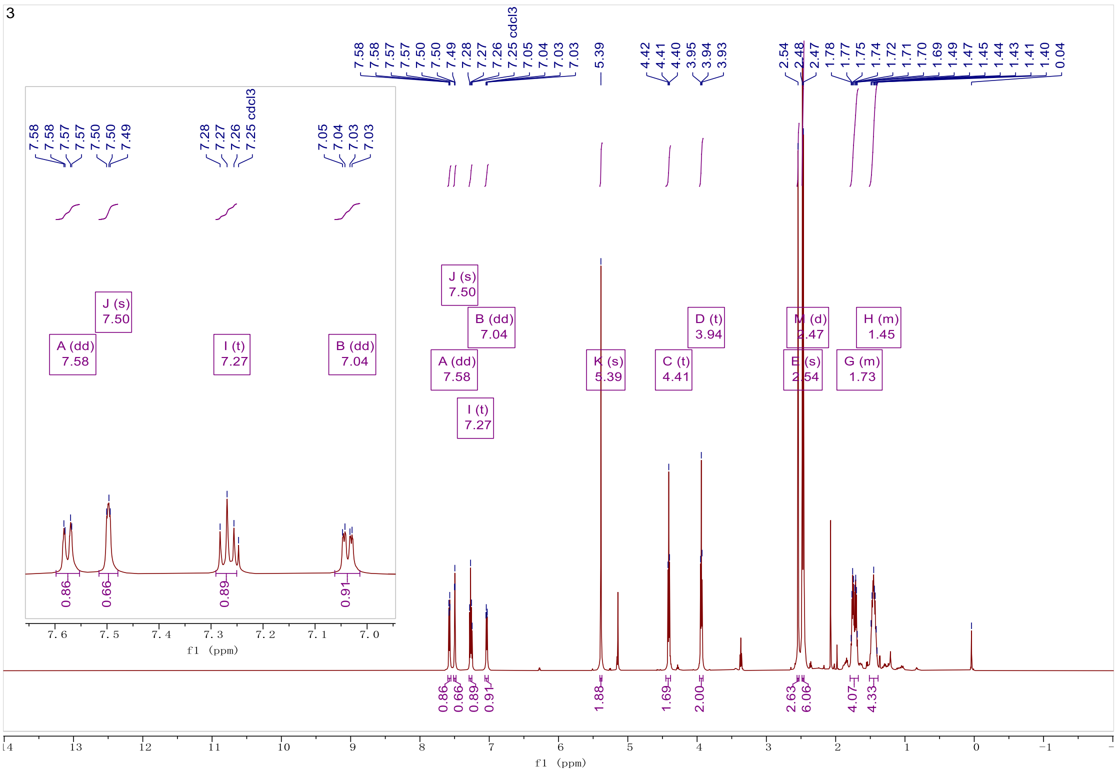

Figure S23. 1H NMR spectrum of (3,5,6-trimethylpyrazin-2-yl)methyl 3-((6-(nitrooxy)hexyl)oxy)benzoate (**15e**)

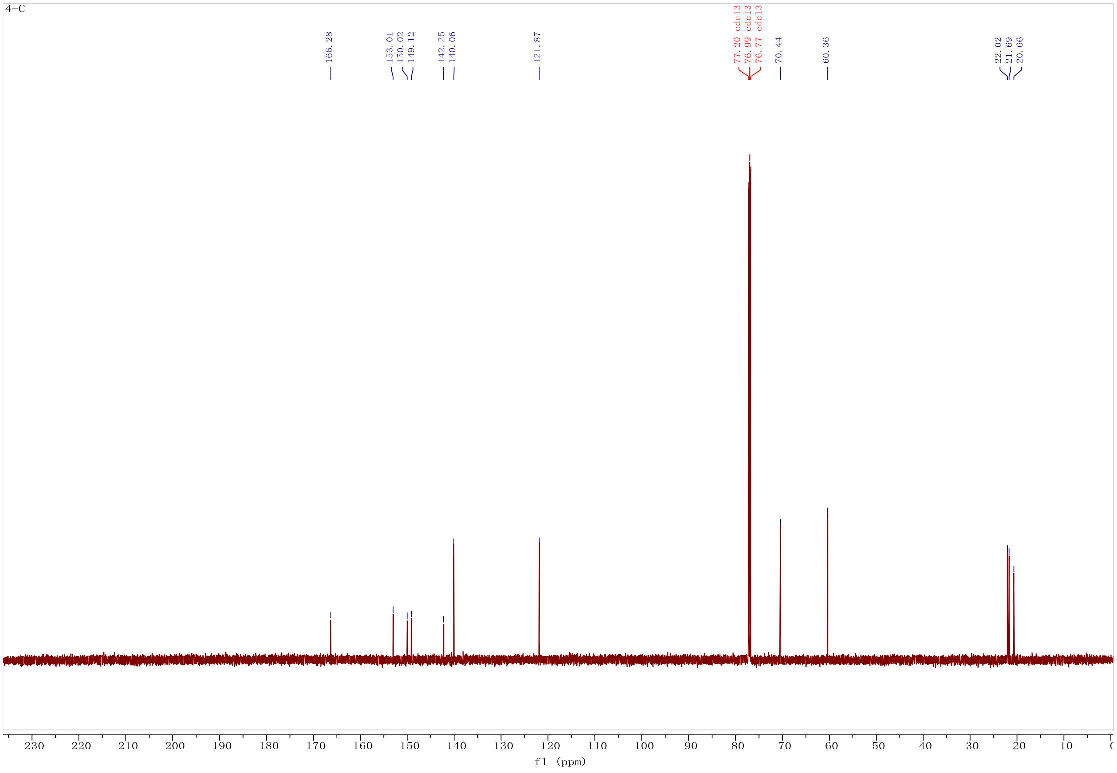

Figure S24. 13C NMR spectrum of 2-(nitrooxy)ethyl (E)-3-(3,5,6-trimethylpyrazin-2-yl)acrylate (**13**)

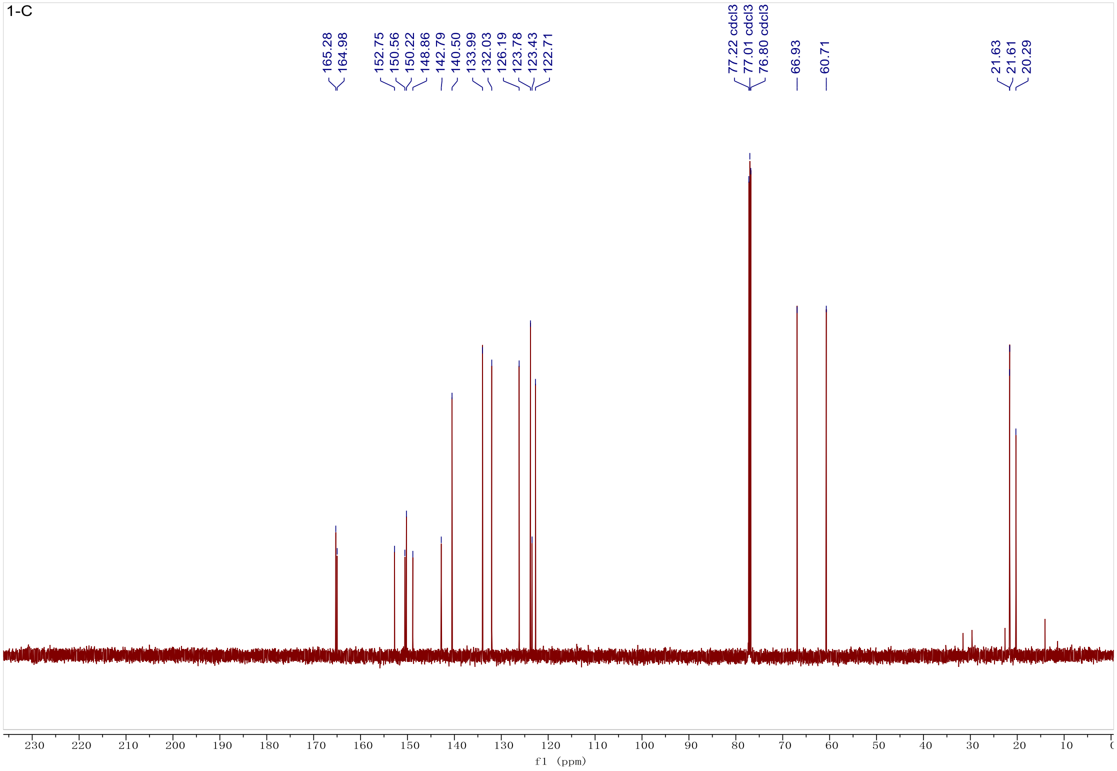

Figure S25. 13C NMR spectrum of 2-(nitrooxy)ethyl (E)-2-((3-(3,5,6-trimethylpyrazin-2-yl)acryloyl)oxy)benzoate (**14a**)

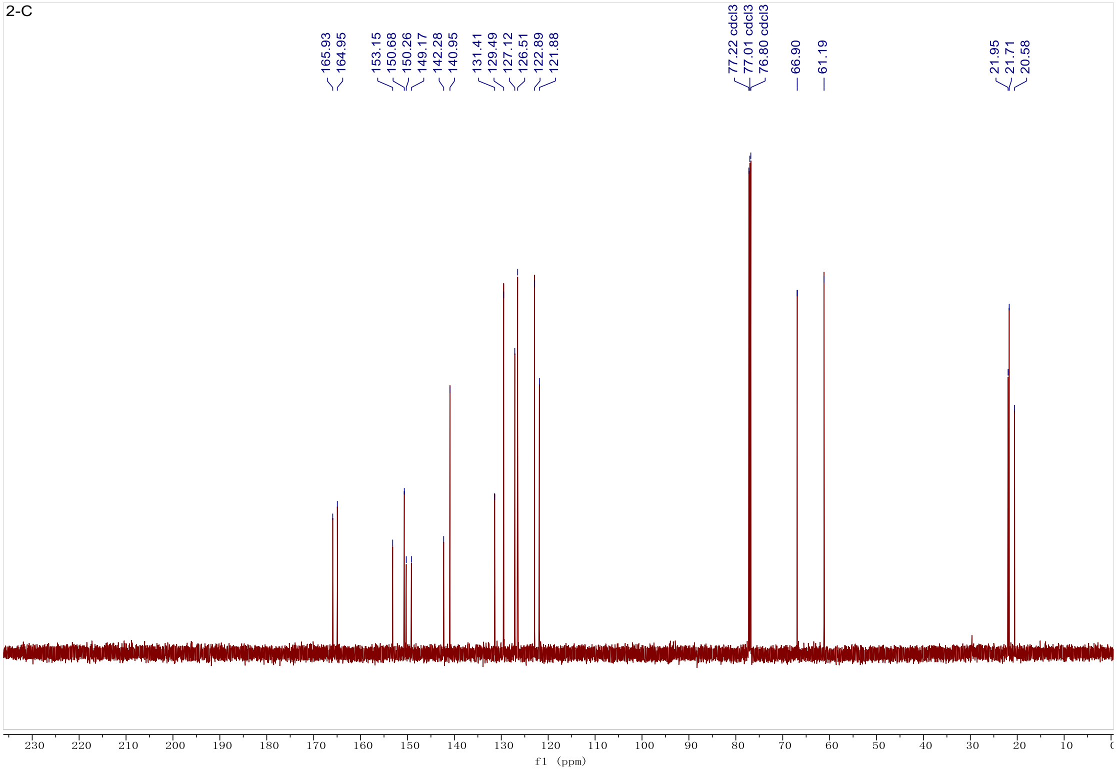

Figure S26. 13C NMR spectrum of 2-(nitrooxy)ethyl (E)-3-((3-(3,5,6-trimethylpyrazin-2-yl)acryloyl)oxy)benzoate (**14b**)

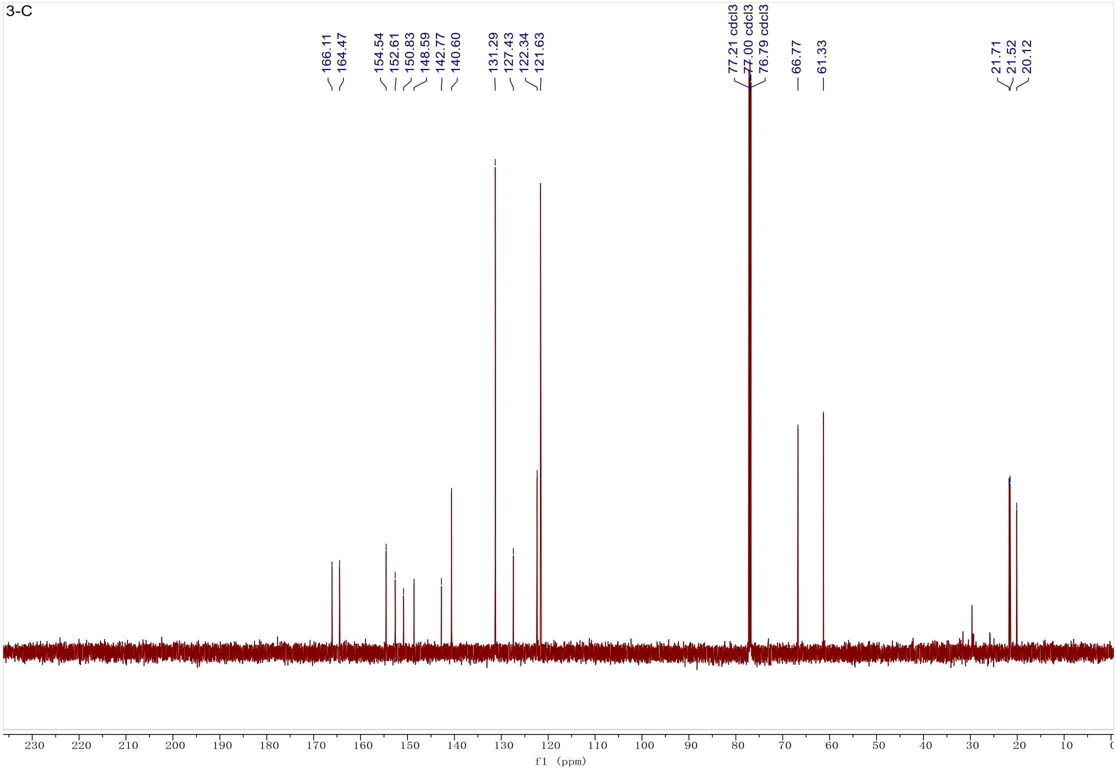

Figure S27. 13C NMR spectrum of 2-(nitrooxy)ethyl (E)-4-((3-(3,5,6-trimethylpyrazin-2-yl)acryloyl)oxy)benzoate (**14c**)

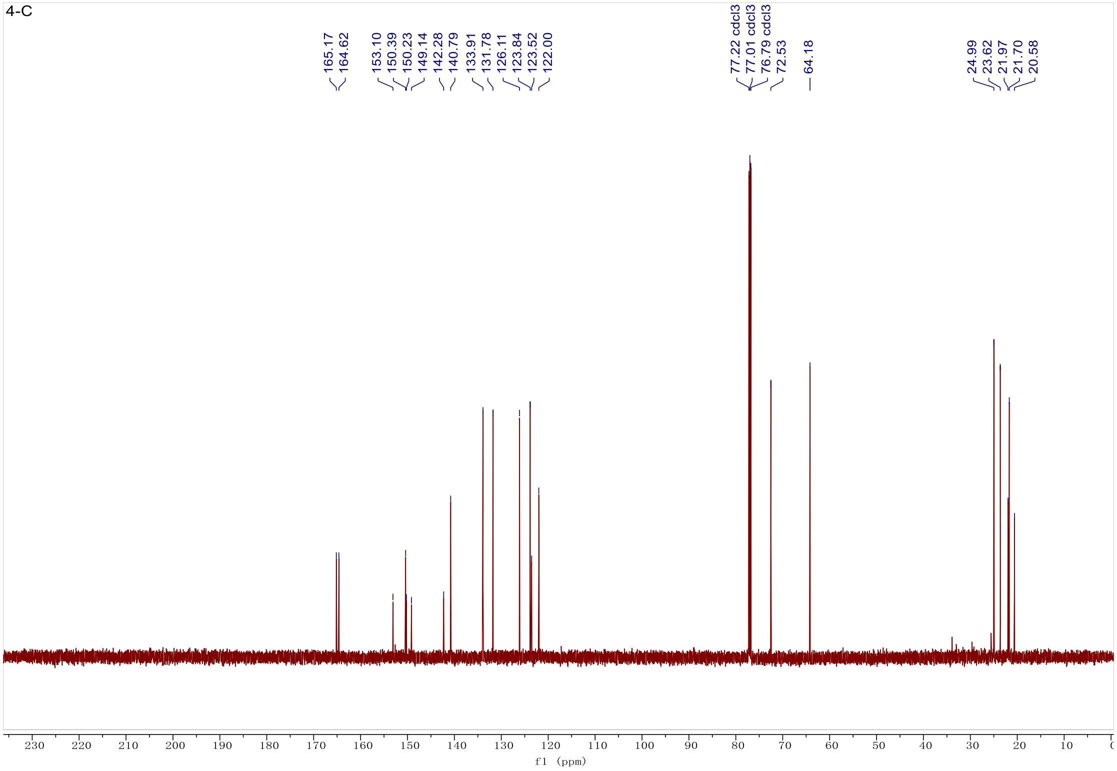

Figure S28. 13C NMR spectrum of 4-(nitrooxy)butyl (E)-2-((3-(3,5,6-trimethylpyrazin-2-yl)acryloyl)oxy)benzoate (**14d**)

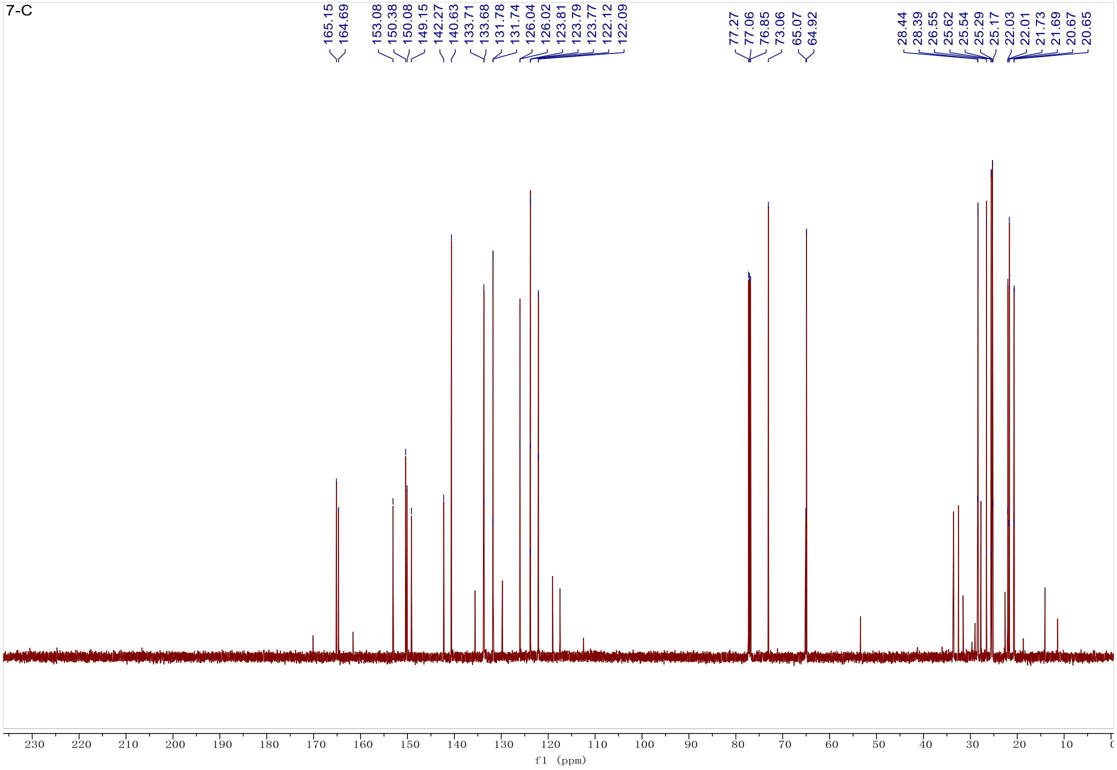

Figure S29. 13C NMR spectrum of 6-(nitrooxy)hexyl (E)-2-((3-(3,5,6-trimethylpyrazin-2-yl)acryloyl)oxy)benzoate (**14e**)

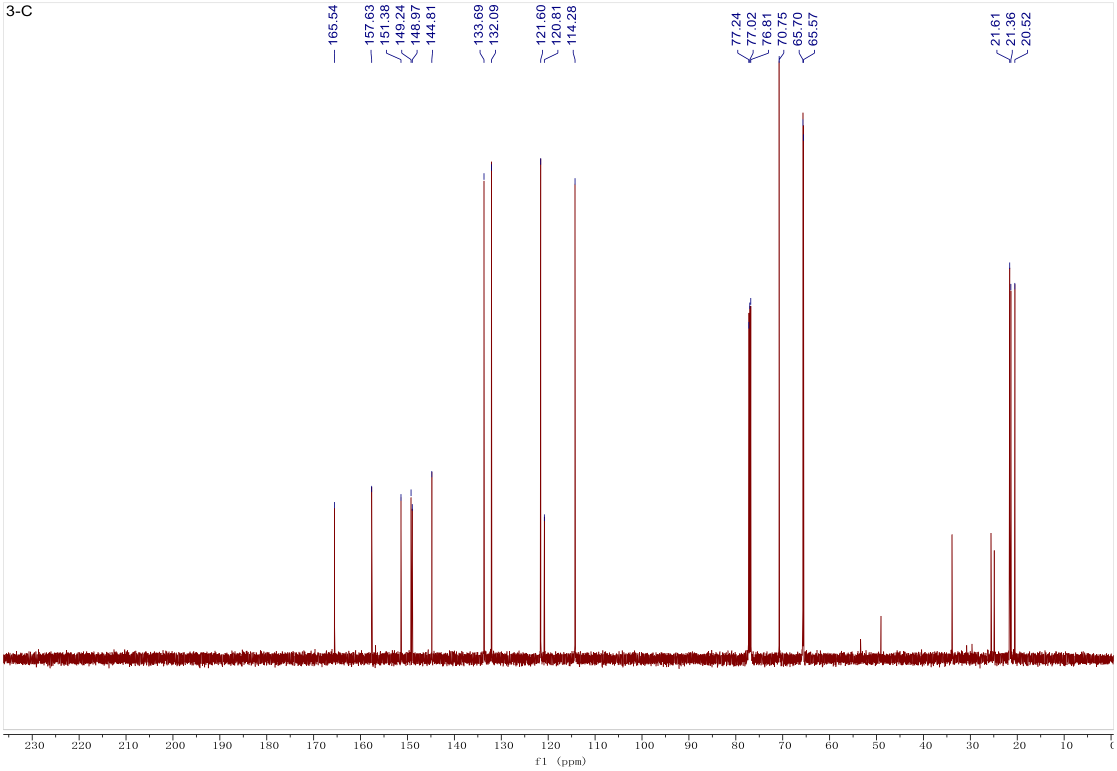

Figure S30. 13C NMR spectrum of (3,5,6-trimethylpyrazin-2-yl)methyl 2-(2-(nitrooxy)ethoxy)benzoate (**15a**)

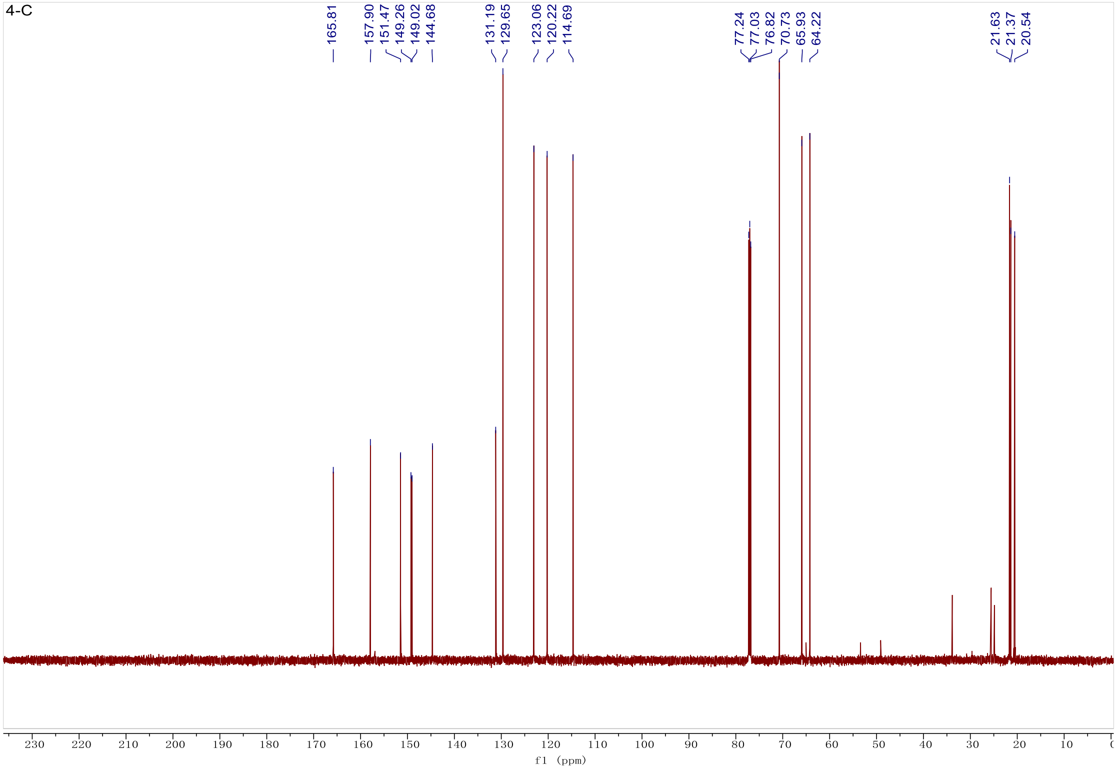

Figure S31. 13C NMR spectrum of (3,5,6-trimethylpyrazin-2-yl)methyl 3-(2-(nitrooxy)ethoxy)benzoate (**15b**)

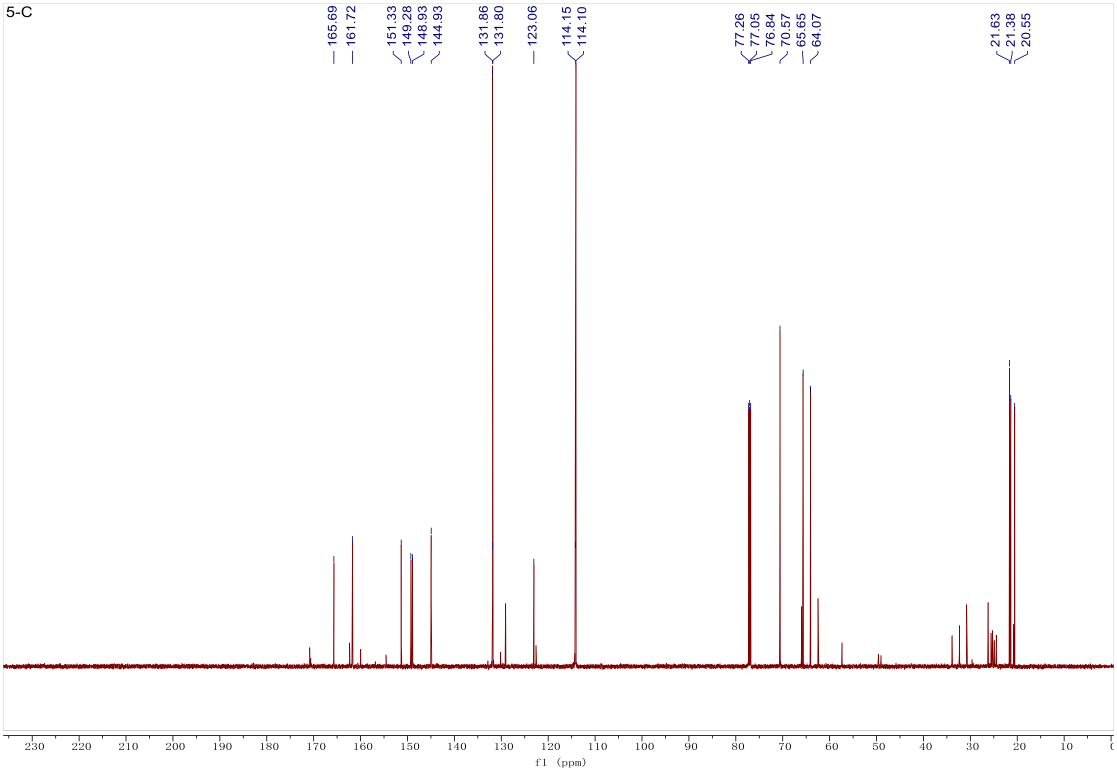

Figure S32. 13C NMR spectrum of (3,5,6-trimethylpyrazin-2-yl)methyl 4-(2-(nitrooxy)ethoxy)benzoate (**15c**)

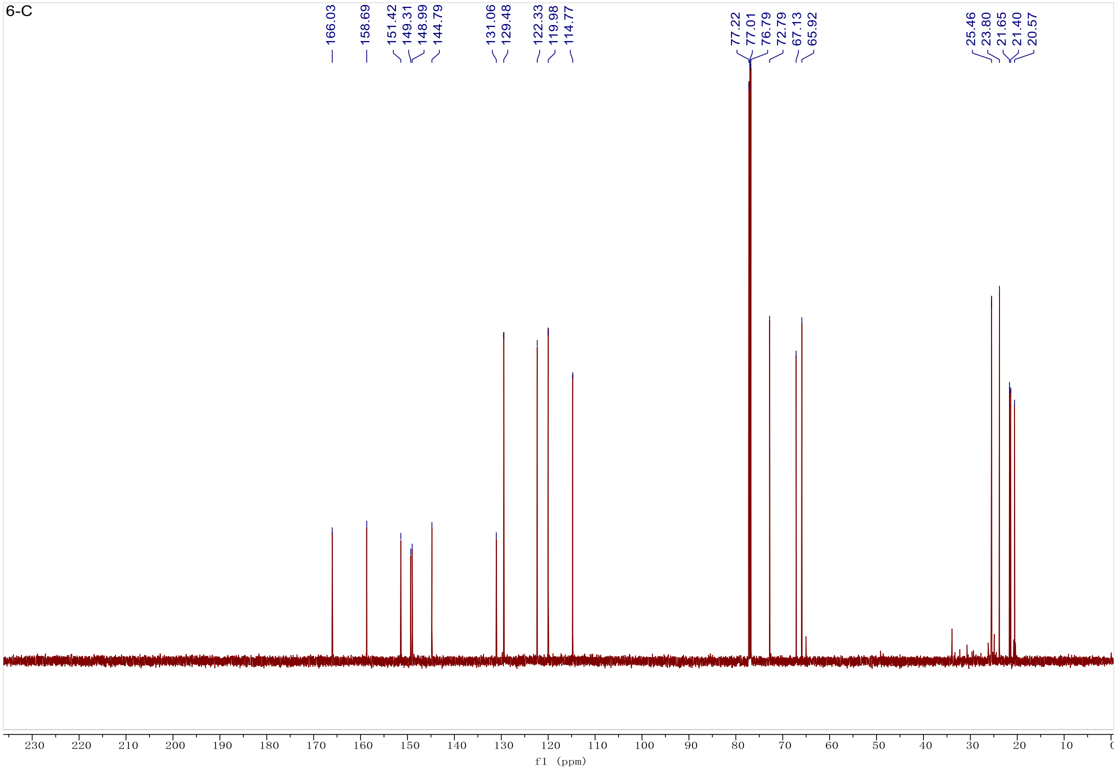

Figure S33. 13C NMR spectrum of (3,5,6-trimethylpyrazin-2-yl)methyl 3-(4-(nitrooxy)butoxy)benzoate (**15d**)

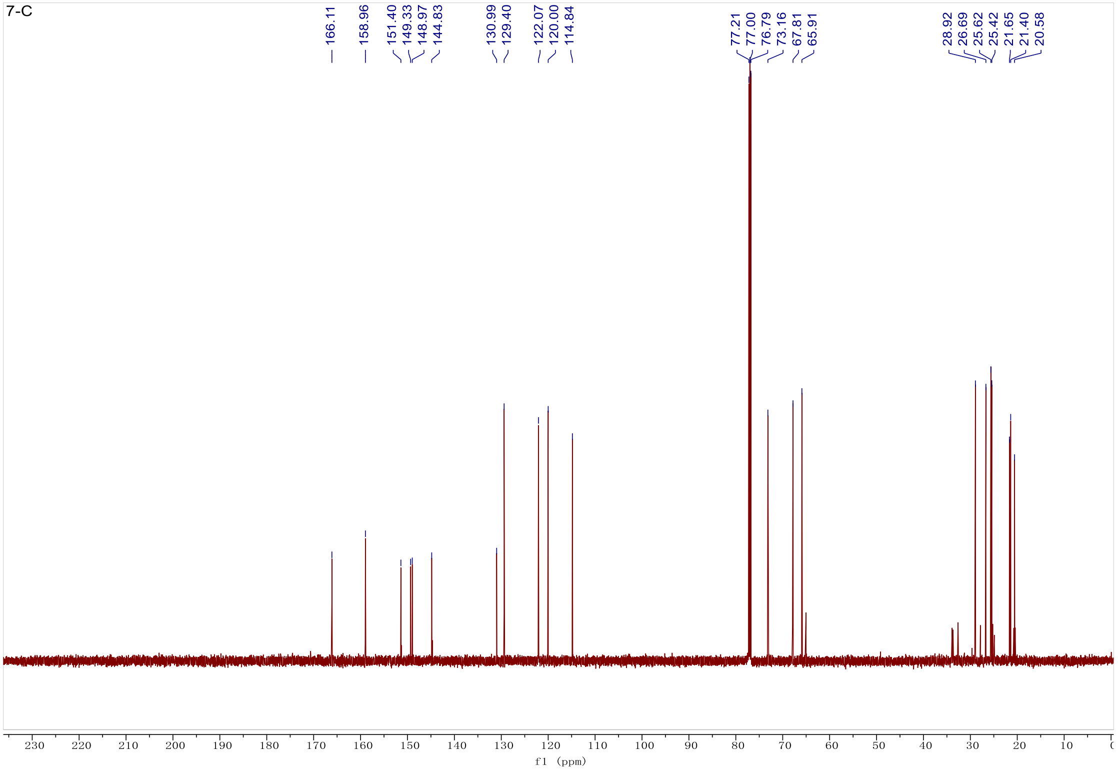

Figure S34. 13C NMR spectrum of (3,5,6-trimethylpyrazin-2-yl)methyl 3-((6-(nitrooxy)hexyl)oxy)benzoate (**15e**)

HRMS spectra of all new compounds.

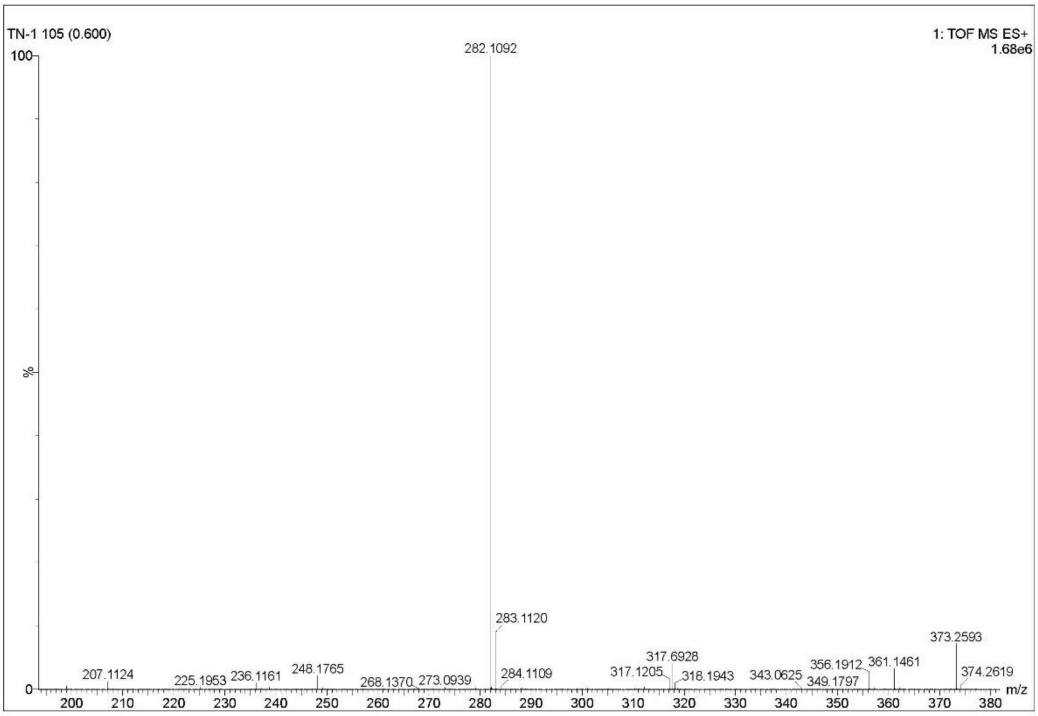

Figure S35. HRMS spectrum of 2-(nitrooxy)ethyl (E)-3-(3,5,6-trimethylpyrazin-2-yl)acrylate (**13**)

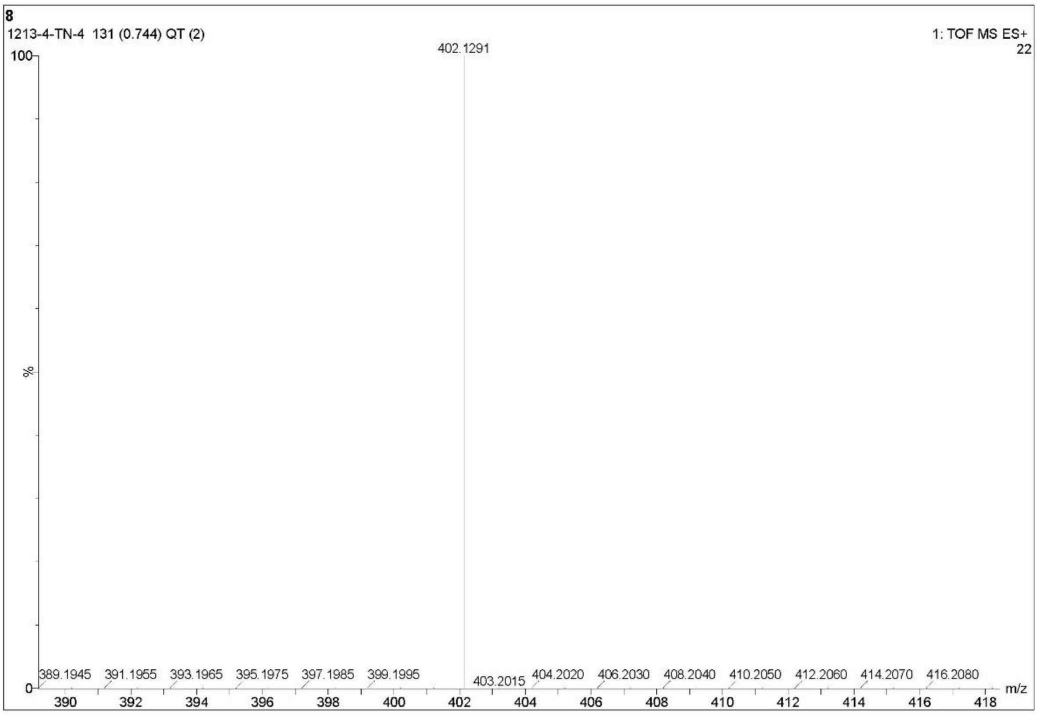

Figure S36. HRMS spectrum of 2-(nitrooxy)ethyl (E)-2-((3-(3,5,6-trimethylpyrazin-2-yl)acryloyl)oxy)benzoate (**14a**)

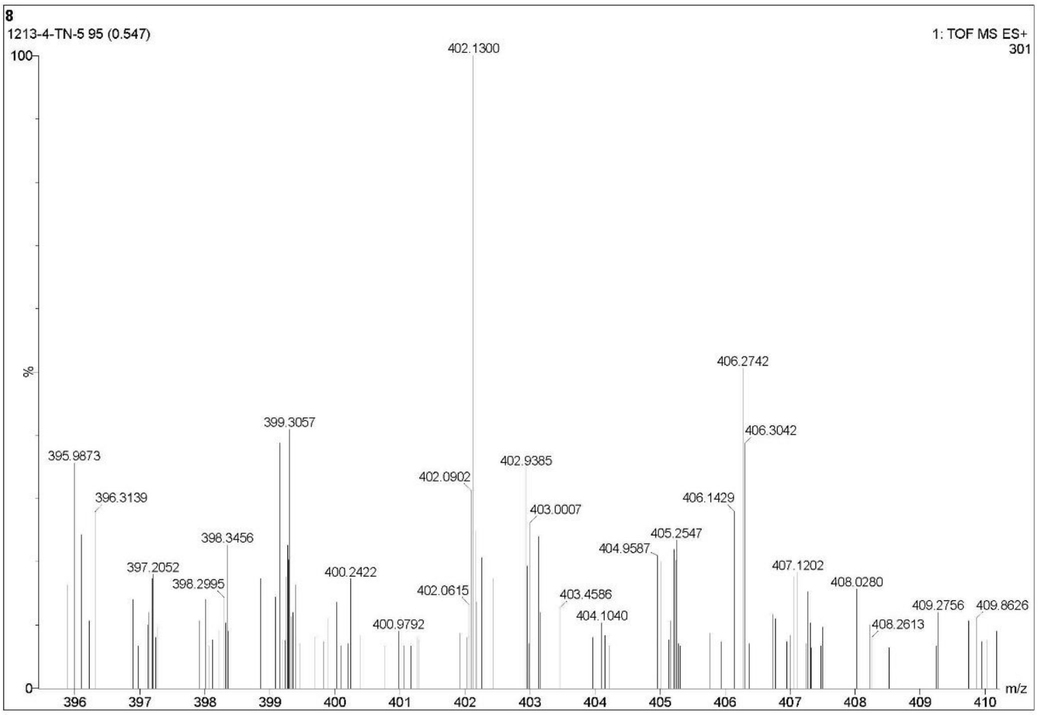

Figure S37. HRMS spectrum of 2-(nitrooxy)ethyl (E)-3-((3-(3,5,6-trimethylpyrazin-2-yl)acryloyl)oxy)benzoate (**14b**)

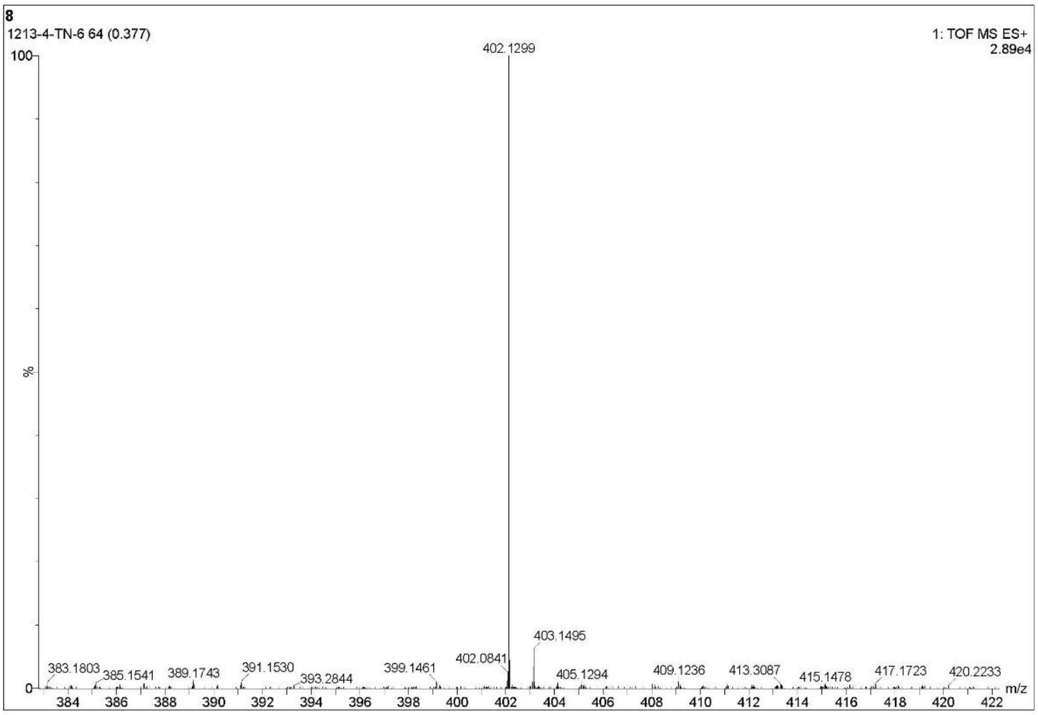

Figure S38. HRMS spectrum of 2-(nitrooxy)ethyl (E)-4-((3-(3,5,6-trimethylpyrazin-2-yl)acryloyl)oxy)benzoate (**14c**)

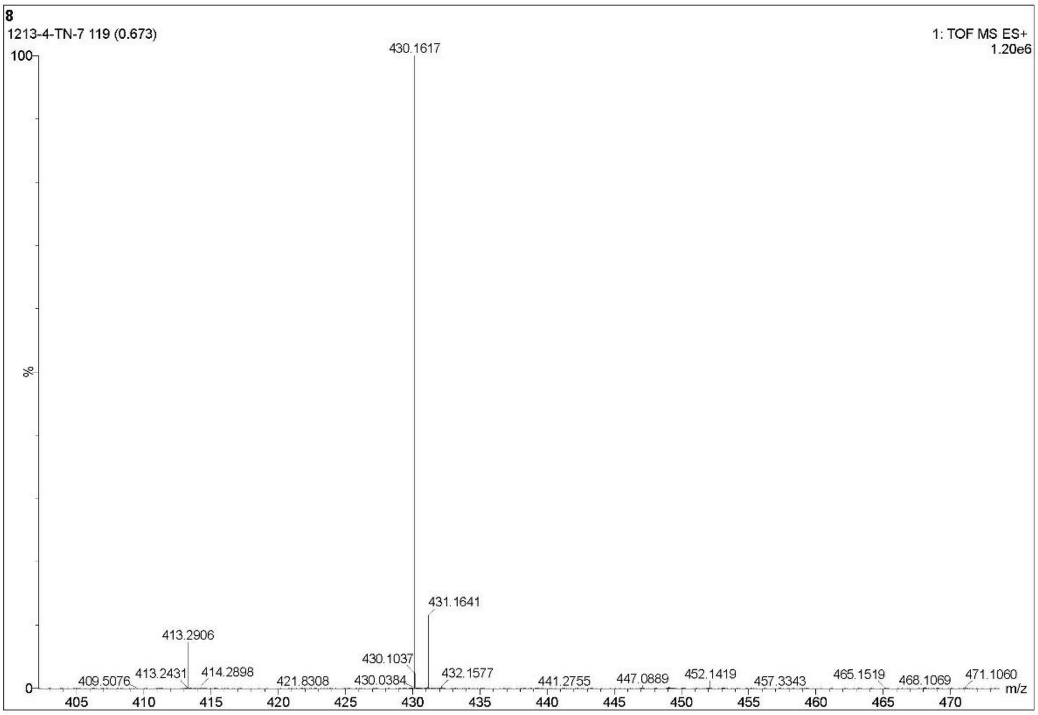

Figure S39. HRMS spectrum of 4-(nitrooxy)butyl (E)-2-((3-(3,5,6-trimethylpyrazin-2-yl)acryloyl)oxy)benzoate (**14d**)

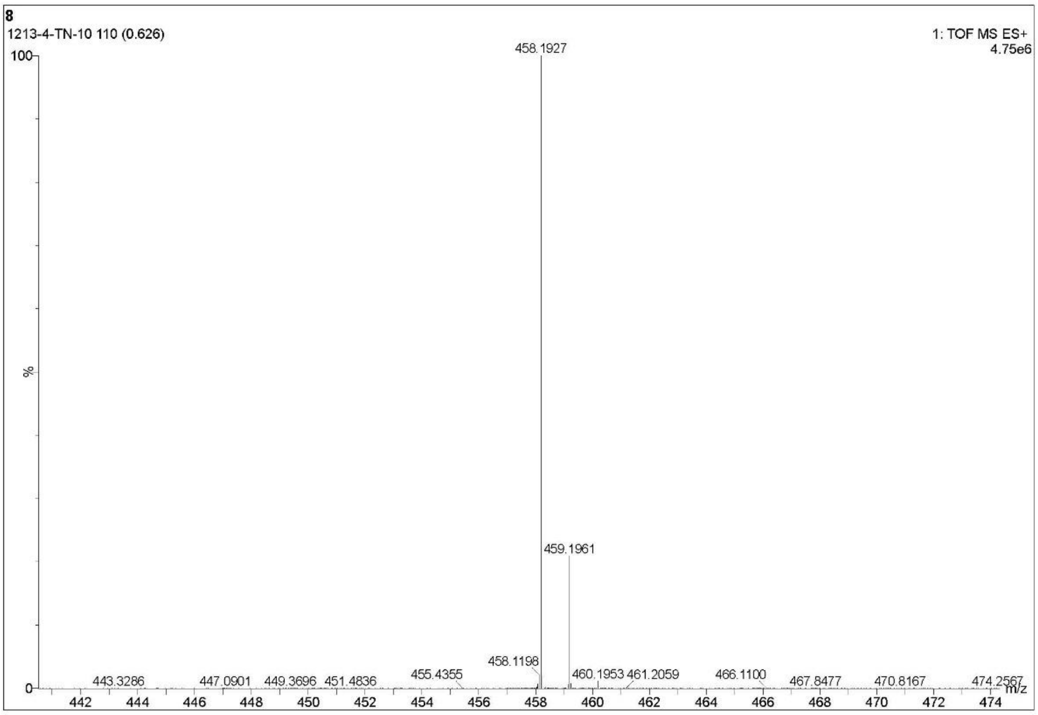

Figure S40. HRMS spectrum of 6-(nitrooxy)hexyl (E)-2-((3-(3,5,6-trimethylpyrazin-2-yl)acryloyl)oxy)benzoate (**14e**)

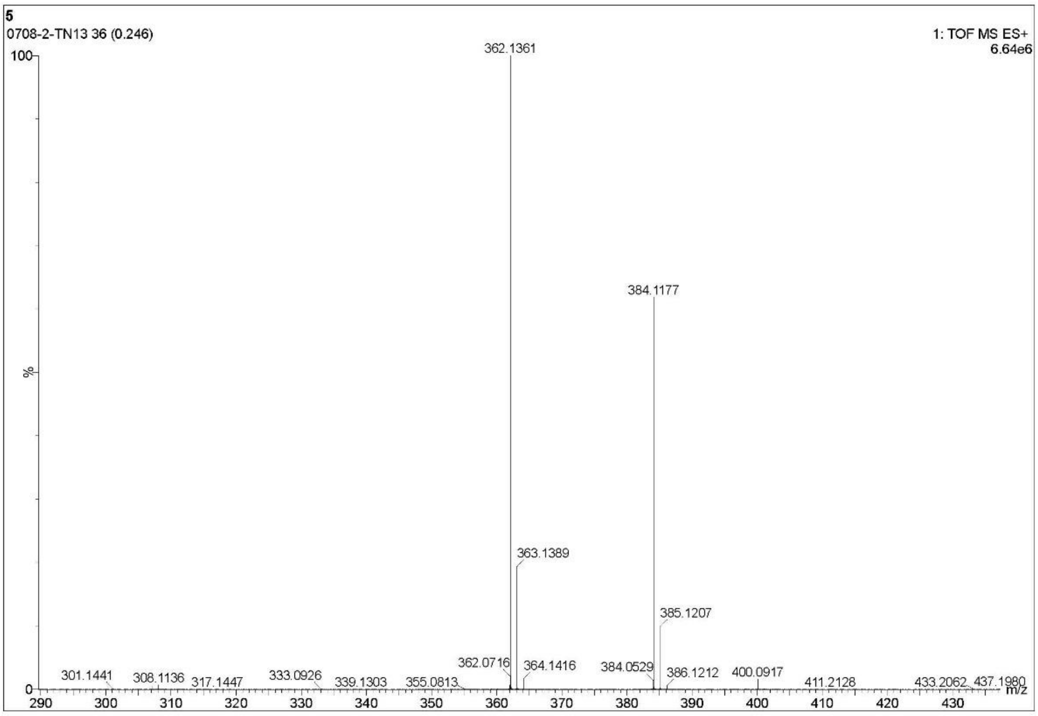

Figure S41. HRMS spectrum of (3,5,6-trimethylpyrazin-2-yl)methyl 2-(2-(nitrooxy)ethoxy)benzoate (**15a**)

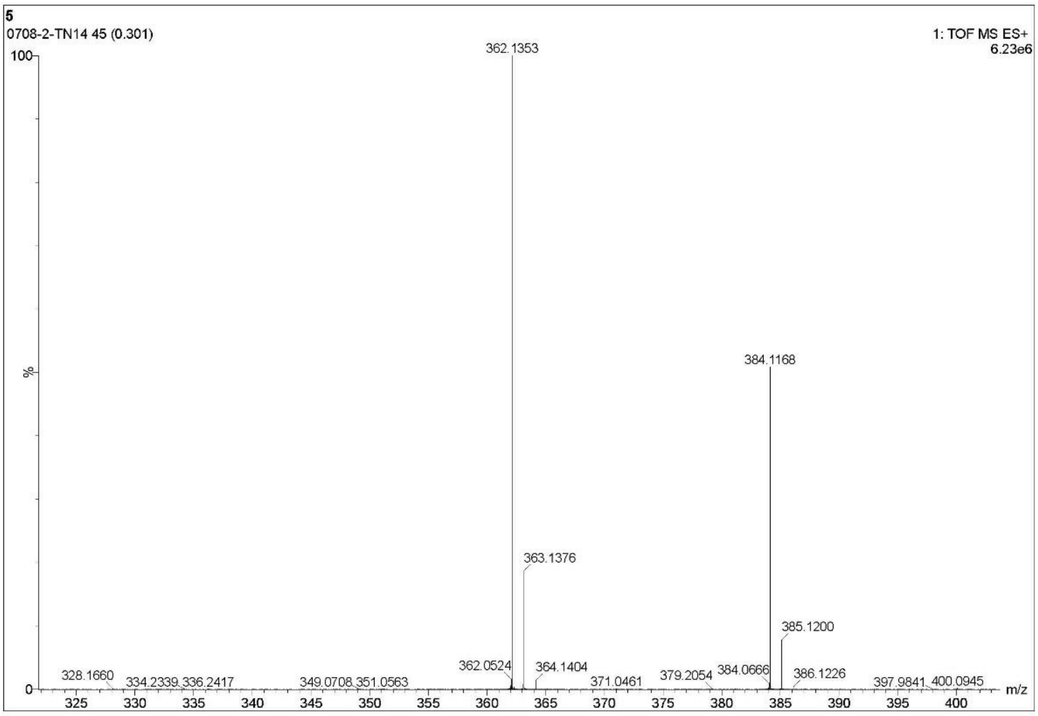

Figure S42. HRMS spectrum of (3,5,6-trimethylpyrazin-2-yl)methyl 3-(2-(nitrooxy)ethoxy)benzoate (**15b**)

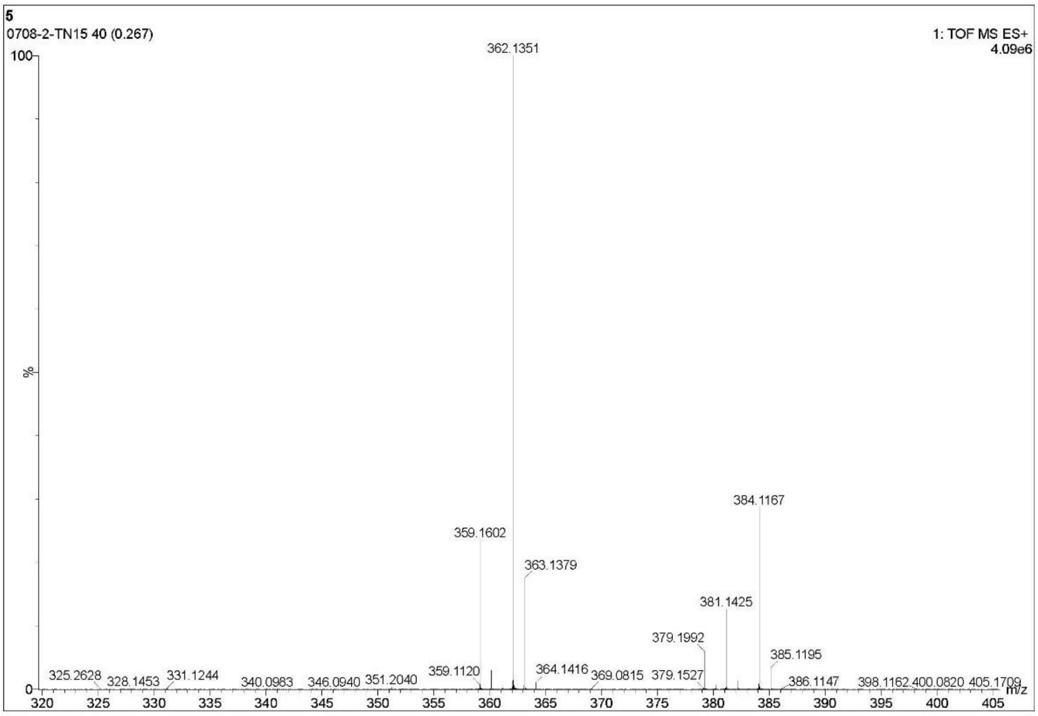

Figure S43. HRMS spectrum of (3,5,6-trimethylpyrazin-2-yl)methyl 4-(2-(nitrooxy)ethoxy)benzoate (**15c**)

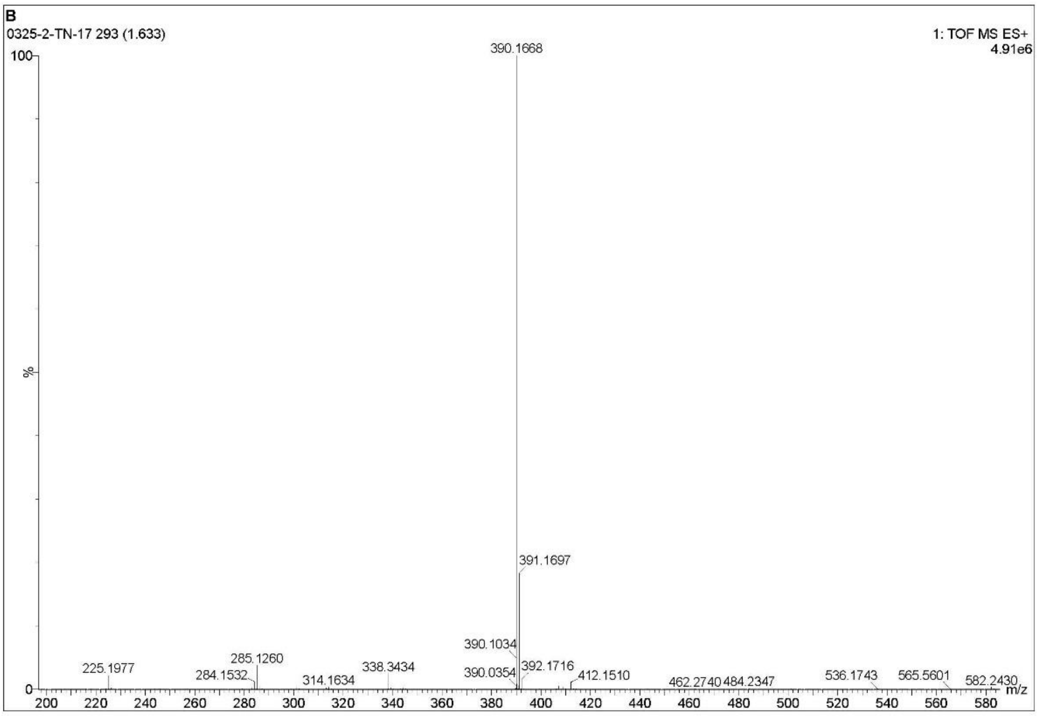

Figure S44. HRMS spectrum of (3,5,6-trimethylpyrazin-2-yl)methyl 3-(4-(nitrooxy)butoxy)benzoate (**15d**)

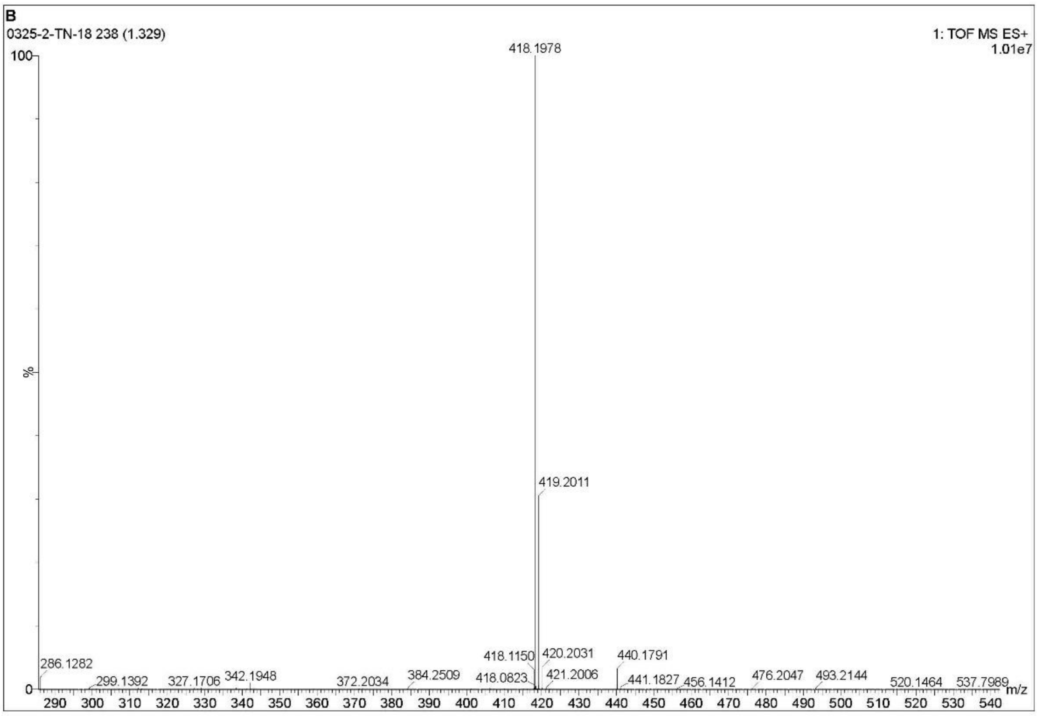

Figure S45. HRMS spectrum of (3,5,6-trimethylpyrazin-2-yl)methyl 3-((6-(nitrooxy)hexyl)oxy)benzoate (**15e**)
